# Identification of different MRI atrophy progression trajectories in epilepsy by subtype and stage inference

**DOI:** 10.1093/brain/awad284

**Published:** 2023-10-09

**Authors:** Fenglai Xiao, Lorenzo Caciagli, Britta Wandschneider, Daichi Sone, Alexandra L Young, Sjoerd B Vos, Gavin P Winston, Yingying Zhang, Wenyu Liu, Dongmei An, Baris Kanber, Dong Zhou, Josemir W Sander, Maria Thom, John S Duncan, Daniel C Alexander, Marian Galovic, Matthias J Koepp

**Affiliations:** Department of Clinical and Experimental Epilepsy, UCL Queen Square Institute of Neurology, London, WC1N 3BG, UK; UCL-Epilepsy Society MRI Unit, Chalfont Centre for Epilepsy, Chalfont St Peter, Buckinghamshire, SL9 0RJ, UK; Department of Neurology, West China Hospital of Sichuan University, Chengdu, Sichuan, 610041, China; Department of Clinical and Experimental Epilepsy, UCL Queen Square Institute of Neurology, London, WC1N 3BG, UK; UCL-Epilepsy Society MRI Unit, Chalfont Centre for Epilepsy, Chalfont St Peter, Buckinghamshire, SL9 0RJ, UK; Department of Neurology, Inselspital, Sleep-Wake-Epilepsy-Center, Bern University Hospital, University of Bern, Bern, Switzerland; Department of Clinical and Experimental Epilepsy, UCL Queen Square Institute of Neurology, London, WC1N 3BG, UK; UCL-Epilepsy Society MRI Unit, Chalfont Centre for Epilepsy, Chalfont St Peter, Buckinghamshire, SL9 0RJ, UK; Department of Clinical and Experimental Epilepsy, UCL Queen Square Institute of Neurology, London, WC1N 3BG, UK; UCL-Epilepsy Society MRI Unit, Chalfont Centre for Epilepsy, Chalfont St Peter, Buckinghamshire, SL9 0RJ, UK; Department of Psychiatry, The Jikei University School of Medicine, Tokyo, 105-8461, Japan; Centre for Medical Image Computing, Departments of Computer Science, Medical Physics, and Biomedical Engineering, UCL, London, WC1E 6BT, UK; Department of Neuroimaging, Institute of Psychiatry, Psychology and Neuroscience, King's College London, London, SE5 8AF, UK; Centre for Medical Image Computing, Departments of Computer Science, Medical Physics, and Biomedical Engineering, UCL, London, WC1E 6BT, UK; Neuroradiological Academic Unit, UCL Queen Square Institute of Neurology, University College London, London, WC1N 3BG, UK; Centre for Microscopy, Characterisation, and Analysis, University of Western Australia, Perth, WA 6009, Australia; Department of Clinical and Experimental Epilepsy, UCL Queen Square Institute of Neurology, London, WC1N 3BG, UK; UCL-Epilepsy Society MRI Unit, Chalfont Centre for Epilepsy, Chalfont St Peter, Buckinghamshire, SL9 0RJ, UK; Department of Medicine, Division of Neurology, Queen’s University, Kingston, K7L 3N6, Canada; Centre for Neuroscience Studies, Queen’s University, Kingston, K7L 3N6, Canada; Department of Neurology, West China Hospital of Sichuan University, Chengdu, Sichuan, 610041, China; Department of Neurology, West China Hospital of Sichuan University, Chengdu, Sichuan, 610041, China; Department of Neurology, West China Hospital of Sichuan University, Chengdu, Sichuan, 610041, China; Centre for Medical Image Computing, Departments of Computer Science, Medical Physics, and Biomedical Engineering, UCL, London, WC1E 6BT, UK; Department of Neurology, West China Hospital of Sichuan University, Chengdu, Sichuan, 610041, China; Department of Clinical and Experimental Epilepsy, UCL Queen Square Institute of Neurology, London, WC1N 3BG, UK; UCL-Epilepsy Society MRI Unit, Chalfont Centre for Epilepsy, Chalfont St Peter, Buckinghamshire, SL9 0RJ, UK; Department of Neurology, West China Hospital of Sichuan University, Chengdu, Sichuan, 610041, China; Stichting Epilepsie Instellingen Nederland – (SEIN), Heemstede, 2103SW, The Netherlands; Department of Clinical and Experimental Epilepsy, UCL Queen Square Institute of Neurology, London, WC1N 3BG, UK; Department of Clinical and Experimental Epilepsy, UCL Queen Square Institute of Neurology, London, WC1N 3BG, UK; UCL-Epilepsy Society MRI Unit, Chalfont Centre for Epilepsy, Chalfont St Peter, Buckinghamshire, SL9 0RJ, UK; Centre for Medical Image Computing, Departments of Computer Science, Medical Physics, and Biomedical Engineering, UCL, London, WC1E 6BT, UK; Department of Clinical and Experimental Epilepsy, UCL Queen Square Institute of Neurology, London, WC1N 3BG, UK; Department of Neurology, Clinical Neuroscience Center, University Hospital Zurich, Zurich, CH-8091, Switzerland; Department of Clinical and Experimental Epilepsy, UCL Queen Square Institute of Neurology, London, WC1N 3BG, UK; UCL-Epilepsy Society MRI Unit, Chalfont Centre for Epilepsy, Chalfont St Peter, Buckinghamshire, SL9 0RJ, UK

**Keywords:** epilepsy, MRI, subtype and stage inference, brain atrophy, disease progression

## Abstract

Artificial intelligence (AI)-based tools are widely employed, but their use for diagnosis and prognosis of neurological disorders is still evolving. Here we analyse a cross-sectional multicentre structural MRI dataset of 696 people with epilepsy and 118 control subjects. We use an innovative machine-learning algorithm, Subtype and Stage Inference, to develop a novel data-driven disease taxonomy, whereby epilepsy subtypes correspond to distinct patterns of spatiotemporal progression of brain atrophy.In a discovery cohort of 814 individuals, we identify two subtypes common to focal and idiopathic generalized epilepsies, characterized by progression of grey matter atrophy driven by the cortex or the basal ganglia. A third subtype, only detected in focal epilepsies, was characterized by hippocampal atrophy. We corroborate external validity via an independent cohort of 254 people and confirm that the basal ganglia subtype is associated with the most severe epilepsy.Our findings suggest fundamental processes underlying the progression of epilepsy-related brain atrophy. We deliver a novel MRI- and AI-guided epilepsy taxonomy, which could be used for individualized prognostics and targeted therapeutics.

## Teaser

Using artificial intelligence algorithms, Xiao *et al.* identify distinct subtypes of spatiotemporal progression of brain atrophy in focal and generalised epilepsies, which variably involve cortex, basal ganglia and hippocampus, and which relate to clinical characteristics.

See Kaestner and Reyes (https://doi.org/10.1093/brain/awad354) for a scientific commentary on this article.

## Introduction

Epilepsy is a common neurological disorder, often chronic and disabling.^1^ The current classification of epilepsy types and syndromes is based on seizure semiology,^2-4^ EEG, aetiology,^5^ imaging^6,7^ and other diagnostic data,^8-10^ which inform treatment and prognosis.^11^ Structural MRI provides reproducible quantitative measures of brain morphology,^12^ which can be conceptualized as biomarkers of pathological processes.^13,14^ In focal epilepsy, cortical thinning encompasses large-scale cortico-subcortical networks.^15^ In idiopathic generalized epilepsy (IGE), structural abnormalities predominantly involve thalamocortical circuitry,^16^ but also extend to fronto-temporo-parietal cortices.^17^ Recent evidence suggests that, in epilepsy, brain atrophy may progress over time.^18^ In cross-sectional studies, brain atrophy in focal epilepsy is related to disease duration, seizure frequency and occurrence of focal-to-bilateral tonic-clonic seizures (FBTCS).^18^ Longitudinal studies highlight widespread progressive grey matter atrophy, affecting regions close to and distant from the key nodes of epileptic networks.^14^ In IGE, grey matter loss is more prominent with longer epilepsy duration and higher seizure frequency.^16,19,20^

The underlying neural processes of progressive brain atrophy remain poorly understood. First, it is uncertain whether there is a consistent spatiotemporal sequence of atrophy progression over time. Second, it remains unclear whether trajectories may vary according to epilepsy types, e.g. focal and generalized epilepsies.^18^ Third, the magnitude and extent of interindividual differences in progression paths and their relationship with clinical characteristics remain undetermined.

Progress may be achieved by applying artificial intelligence (AI), particularly techniques in the machine learning subfield, that are increasingly used in biomedical research.^21^ In epilepsy, imaging-based machine learning has successfully lateralized temporal lobe epilepsy (TLE),^22^ identified radiologically occult epileptogenic lesions,^23,24^ predicted epilepsy surgery outcomes,^25^ and typified individual-specific patterns of whole-brain structural reorganization relating to disease severity.^26^

Here we use an unsupervised and data-driven machine learning algorithm, Subtype and Stage Inference (SuStaIn),^27^ recently developed to capture patterns of disease progression in chronic conditions, particularly neurodegenerative disorders such as Alzheimer's and Parkinson’s disease.^27-31^ SuStaIn reaches longitudinal inference from cross-sectional data. Specifically, it automatically identifies distinct spatiotemporal trajectories (patterns) of cumulative pathological alteration shown by measured biomarkers and quantifies their level of individual co-expression.^27,31,32^ Our study employs SuStaIn to decode individualized signatures of progressive cortico-subcortical atrophy in large focal and generalized epilepsy cohorts. We introduce a novel, machine learning-guided and MRI-based epilepsy taxonomy that combines categorical and dimensional perspectives by (i) quantifying main progression patterns of grey matter atrophy in each individual; and (ii) identifying subgroups based on the dominant progression pattern. We also replicate our findings in an external validation cohort and relate machine learning-identified subtypes to clinical characteristics.

## Materials and methods

### Participants

Our study assessed participants from two separate cohorts. The discovery cohort consisted of structural MRI data of a single-centre dataset involving long-term follow-up^33^ of individuals with focal epilepsy or IGE, investigated with 3 T high-resolution MRI (GE) at the Chalfont Centre for Epilepsy (UCL Queen Square Institute of Neurology/National Hospital of Neurology and Neurosurgery, London), UK, between January 2004 and March 2018. An external, independent validation cohort included individuals scanned with 3 T high-resolution MRI at the Department of Neurology of West China Hospital, Sichuan University, Chengdu, China, between June 2013 and December 2020. Before MRI data preprocessing and quality control (Supplementary material), 894/211 people with epilepsy (discovery/validation cohorts) and 121/73 control participants (discovery/validation cohorts), for a total of 1299 individuals, were considered for inclusion in this study.

For both cohorts, diagnosis, lateralization and localization of focal epilepsy were confirmed by a multidisciplinary epilepsy team, based on clinical history, neurological examination, seizure semiology, ambulatory EEG monitoring, interictal and ictal EEG during long-term video-EEG telemetry, structural MRI, ^18^F-fluorodeoxyglucose PET (in a subset), and neuropsychological assessments. People with brain lesions other than hippocampal sclerosis, those with poor MRI data quality, and those without adequately detailed clinical data were not included in this study. People with IGE had a typical clinical history and a previous routine EEG with interictal generalized (poly-)spike-and-wave discharges at UCL (discovery cohort) or Sichuan University (validation cohort). All participants had a clinical structural MRI scan as part of diagnostic investigations. Clinical characteristics were collected through a review of the entry in medical notes closest in time to the date of the MRI scan. The number of life-time trialled anti-seizure medications (ASM) and the duration of epilepsy were recorded on the day of the MRI scan. Complete details are provided in Table 1. Control subjects from both sites were recruited from the local community and had no family history of epilepsy or neurological or psychiatric disorders.

**Table 1:** Demographic and clinical characteristics of discovery and validation cohorts

	Discovery cohort			Validation cohort		
	Focal epilepsy	IGE	Healthy controls	Focal epilepsy	IGE	Healthy controls
Participant number	503	193	118	122	61	71
Sex, female/male	248/255	112/81	74/44	47/75	27/34	39/32
Age, mean (SD)	35.0 (10.8)	33.7 (11.4)	36.3 (12.4)	25.1 (8.6)	20.6 (5.6)	26.1 (6.9)
Handedness	R (433)	R (191)	R (108)	R (121)	R (61)	R (71)
	L (63)	L (2)	L (10)	L (1)		
	A (7)					
Age of onset, mean (SD)	15.6 (12.2)	13.9 (6.1)^a^	N/A	15.2 (10.3)	14.0 (3.0)	N/A
Duration of epilepsy mean (SD)	19.9 (12.6)	20.5 (12.2)^a^	N/A	9.9 (7.2)	5.0 (3.1)	N/A
Lateralization of seizure focus (*n*)	L (225)	N/A	N/A	L (83)	N/A	N/A
	R (193)			R (32)		
	B (44)			B (4)		
	U (41)			U (3)		
Localization of seizure focus, or epilepsy type (*n*)	TLE (328)	JAE (43)	N/A	TLE (50)	JME (61)	N/A
	FLE (88)	JME (46)		FLE (50)		
	PLE (27)	GTCS-unc (104)		PO (22)		
	OLE (5)					
	U (55)					
Localization of seizure focus of cases with proven lateralization (*n*)	TLE (281)	N/A	N/A	TLE (50)	N/A	N/A
	FLE (81)			FLE (45)		
	PLE (24)			PO (20)		
	OLE (4)					
	U (28)					
Unilateral focal epilepsy (%)	418 (83.1)	N/A	N/A	115 (94.3)	N/A	N/A
FBTCS (focal), GTCS (generalized),^b^ % of patients	59.6	53.9	N/A	62.4	35.9	N/A
HS, *n*	96	N/A	N/A	34	N/A	N/A
Seizure frequency, median (range)	3 (0–4)	1 (0–4)	N/A	2 (0–4)	2 (0–4)	N/A
ASM, median (range)	2 (0–6)	2 (0–4)	N/A	2 (0–3)	1 (0–2)	N/A
Surgery, *n* (%)	108 (21.5%)			34 (27.9%)		

ASM = anti-seizure medication; B = bilateral; FBTCS = focal to bilateral tonic-clonic seizure; FLE = frontal lobe epilepsy; GTCS = generalized tonic clonic seizure; GTCS-unc = IGE unclassified, with GTCS as primary seizure subtype; HS = hippocampal sclerosis; IGE = idiopathic generalized epilepsy; JAE = juvenile absence epilepsy; JME = juvenile myoclonic epilepsy; L = left; *n* = number; OLE = occipital lobe epilepsy; PLE = parietal lobe epilepsy; PO = posterior quadrant; R = right; SD = standard deviation; TLE = temporal lobe epilepsy; U = undetermined.

Reliable data pertaining to age at seizure onset and duration of epilepsy were available in 129 individuals of the discovery cohort with IGE.

At least one FBTCS (focal epilepsies) or GTCS (IGE) experienced in the year before the MRI.

### Protocol approvals, registrations and participant consent

This study was pursued under a protocol approved by the UCL and University College London Hospital Joint Research Ethics Committee (20/LO/0149). It involved an analysis of previously acquired clinical data posing no risk to people requiring individual consent. The UCL and University College London Hospital Joint Research Ethics Committee approved recruiting healthy controls as part of previous studies. Written informed consent was obtained from healthy participants per the Declaration of Helsinki standards. The West China Hospital Clinical Trials and Biomedical Ethics Committee approved participant recruitment for the validation cohort. All participants provided written informed consent by the standards of the Declaration of Helsinki.

### MRI data acquisition

Participants from UCL were scanned between January 2004 and March 2018. For those scanned between January 2004 and March 2013 (all people with IGE, 336 people with focal epilepsy and 50 control subjects) (Supplementary material, ‘Methods’ section), MRI data were acquired on a 3 T GE Signa HDx scanner with a coronal T_1_-weighted 3D inversion recovery fast spoiled gradient echo (IR-FSPGR) sequence, repetition time/echo time/inversion time: 8.1/3.1/450 ms, voxel size: 0.9 × 0.9 × 1.1 mm. For those scanned between March 2013 and March 2018 (355 people with focal epilepsy and 71 healthy control subjects) (Supplementary material, ‘Methods’ section), MRI data were acquired on a 3 T GE Discovery MR750 scanner using a 3D T_1_-weighted magnetization prepared rapid acquisition gradient echo (MPRAGE) sequence with echo time/repetition time/inversion time: 3.1/7.4/400 ms, voxel size: 1.0 × 1.0 × 1.0 mm. For participants in the external validation cohort, MRI data were acquired at the West China Hospital between June 2013 and December 2020, using a 3 T Siemens Tim Trio MRI scanner with an eight-channel head coil. High-resolution T_1_-weighted MRI was acquired using a 3D MPRAGE sequence with repetition time/echo time/inversion time: 1900/2.6/900 ms, voxel size: 1.0 × 1.0 × 1.0 mm.

### MRI data preprocessing

To evaluate brain atrophy, we focused on cortical thickness as an established, surface-based marker of cortical morphology, that reflects cellular-level features including size, density and arrangement of neurons, glia and nerve fibres.^34,35^ To this end, we employed the Computational Anatomy Toolbox (CAT12) running in Statistical Parametric Mapping 12 (SPM12) and MATLAB 2021a (Mathworks).^36,37^ Cortical thickness was estimated using the projection-based thickness method, previously validated using spherical and brain phantoms confirming accurate measurements under a comprehensive set of parameters for several thickness levels.^36^ The CAT12 toolbox provided excellent test-retest reliability (*R*^2^ = 0.986) and was validated against other cortical surface reconstruction methods, showing fewer measurement errors than similar software.^37,38^ We used an inverse-consistent longitudinal surface registration approach implemented in CAT12 to prevent an asymmetry bias from arising when data from multiple time points are analysed. All data were controlled for quality according to standardized CAT12 pipelines; scans that exhibited misalignment, misregistration or inaccurate thickness estimation were excluded. Image quality ratings were estimated by scaling image noise, inhomogeneities and resolution to a single score within the CAT12 quality assurance framework. Following data quality checks (Supplementary material, ‘Methods’ section), 503/122 participants with focal epilepsy, 193/61 people with IGE and 118/71 healthy control participants in the discovery/validation cohorts, for a total of 814 individuals, were retained for analysis. Detailed demographic and clinical characteristics of these individuals are provided in Table 1.

To obtain measures of hippocampal volume, we employed Hipposeg (http://niftyweb.cs.ucl.ac.uk/program.php? p=HIPPOSEG), an open-source, multi-atlas-based, previously validated hippocampal segmentation algorithm.^39,40^ Hipposeg delineates the hippocampus with no more variability than seen between expert human raters, and is robust to hippocampal morphological alterations, including atrophy. It was built using 876 3 T and 202 1.5 T scans of people with epilepsy. It has continuously improved and demonstrated superior delineation of diseased hippocampi compared to other automated segmentation methods.^41,42^ Volumes of other subcortical structures relevant in epilepsy, including the thalamus, amygdala, caudate, putamen and globus pallidus, and total intracranial volume, were extracted using a parcellation algorithm based on Geodesic Information Flows (GIF),^43^ freely available within NiftyWeb (http://cmictig.cs.ucl.ac.uk/niftyweb, UCL Centre for Medical Image Computing, UK). Adjustment for total intracranial volume is described later. Previous work showed excellent agreement between GIF-derived subcortical volumes and those obtained using FSL-FIRST,^44^ and between GIF-derived cortical volumes and those obtained using SPM12^45^ as used previously in the study of neurodegenerative diseases^44,46,47^ and in previous SuStaIn studies for analysing neurodegenerative disorders.^31,32,44^

### Specification of regions of interest

**Figure 1:**
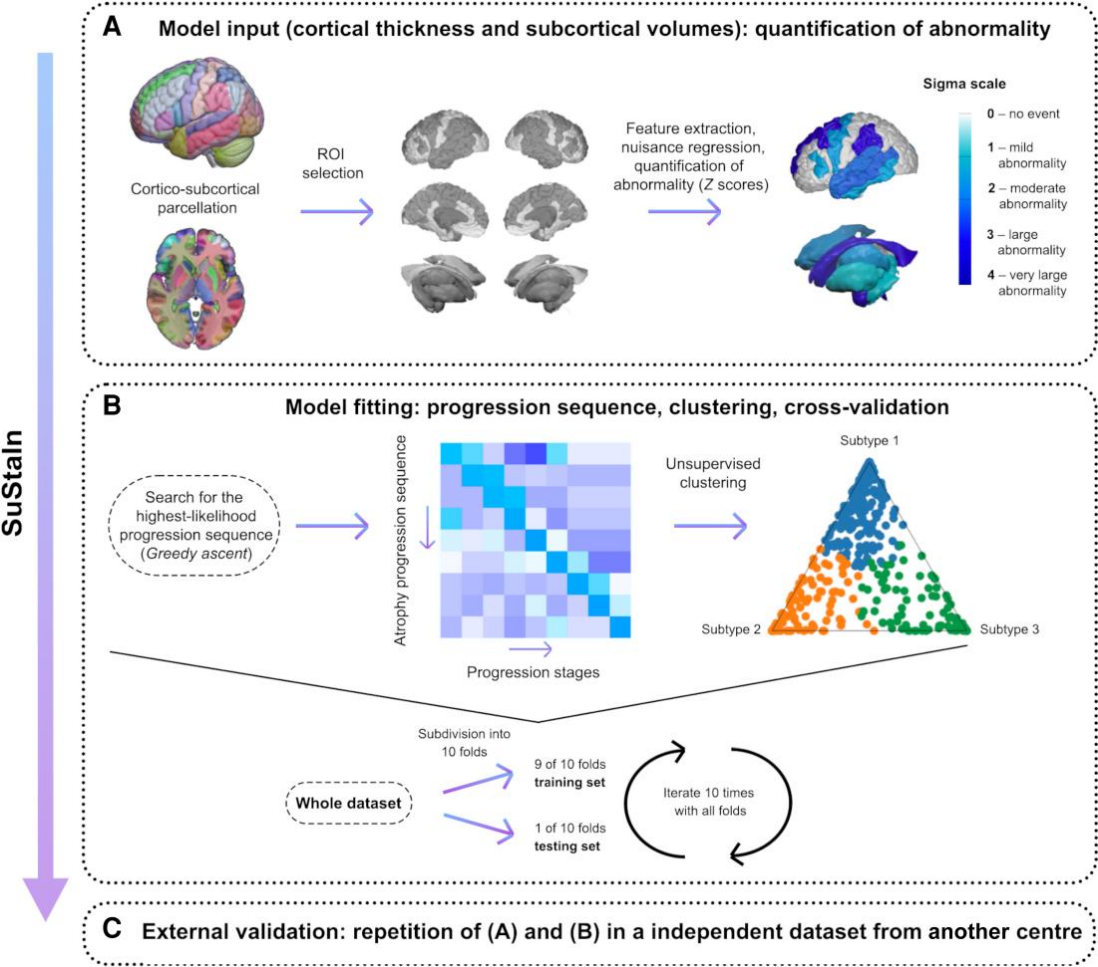
**Visual schematic of the SuStaIn event-based model.** We applied the SuStaIn algorithm to derive spatiotemporal patterns of progression of atrophy in large samples of people with focal epilepsy and IGE (*n* = 1299). The three main steps of the algorithm consist of: (**A**) Model input: selection of regions of interest, adjustment for nuisance variables, and conversion of regional grey matter metrics into *z*-scores relative to healthy control data; (**B**) Model fitting: computation of the best-fit probability distributions for normal and atrophic brain regions, identification of the most likely progression sequence, and quantification of uncertainty with cross-validation. An illustrative positional variance diagram, displayed on the left-hand side, shows an example of an atrophy progression sequence with the highest likelihood on the *y-*axis, and the number of model stages (i.e. sequence positions) on the *x*-axis; the intensity of each entry corresponds to the proportion of Markov Chain Monte Carlo samples for which a certain region of the *y*-axis appears at the respective stage of the *x*-axis. An exemplary ternary plot shows the probability with which each individual is assigned to a given subtype, whereby each vertex represents the point at which membership of a given subtype is maximal (100%). The dots correspond to individual data and are labelled by final subtype classification: subtype 1, subtype 2 or subtype 3. (**C**) External validation: repetition of procedures detailed in passages **A** and **B** for the external validation cohort, to address generalizability.

Based on the recent international multicentre ENIGMA-epilepsy structural MRI study,^18^ we selected the following bilateral regions of interest (ROI) from the Desikan-Killiany (DK40) atlas: (i) 28 cortical ROIs: left and right superior frontal gyrus, caudal middle frontal gyrus, inferior frontal gyrus—pars triangularis, precentral gyrus, paracentral lobule, superior temporal gyrus, transverse temporal gyrus, middle temporal gyrus, inferior temporal gyrus, supramarginal gyrus, precuneus, posterior cingulate cortex, lingual gyrus, and cuneus; and (ii) 12 mesiotemporal and subcortical ROIs, including left and right hippocampus, amygdala, thalamus, and basal ganglia structures, including caudate, globus pallidus and putamen (Fig. 1A, Supplementary Table 1 and Supplementary Fig. 1). While the above parcellation scheme was constrained to a maximum of 40 ROIs and did not allow for whole-brain inference, as in prior work with SuStaIn,^27^ the selection of ROIs for our study was attained to maximize the trade-off between accuracy and computational tractability, and was motivated *a priori* by the findings of large-scale multicentre studies of the ENIGMA-epilepsy consortium,^17,18^ which provided a state-of-the-art characterization of the spatial distribution of grey matter alterations in focal epilepsy and IGE.

As in prior structural MRI investigations employing the SuStaIn algorithm,^31,32^ we adjusted ROI-wise cortical thickness within each cortical ROI for age at scan and (binary) sex, and adjusted mesiotemporal and subcortical volumes for total intracranial volume, age at scan, and sex; specifically, we constructed a linear regression model for each region separately, entering the value of cortical thickness and subcortical volumes as the dependent variable and the variables mentioned above as predictors, and retained the unstandardized residuals (of the fit) for each region for subsequent analyses. For each of the 40 MRI measures listed above, we combined the two healthy control datasets to fit a Bayesian linear regression model with total intracranial volume, sex, age and age squared as independent variables, and each MRI measure as the outcome.^27,31,32^ As previously reported,^27,31,32^ we computed the expected values using this model and subtracted the observed values to obtain residual values of each MRI variable. We refer to the residual values as adjusted values.^27,31,32^ To investigate the effects of seizure focus laterality in people with focal epilepsies, we conducted a subgroup analysis by including only people with proven lateralization of the seizure focus and regrouping regions into ‘ipsilateral’ or ‘contralateral’.

### SuStaIn

As in previous neurological studies,^27,29,32^ we employed the SuStaIn algorithm^27^ to identify distinct patterns of spatiotemporal progression from cross-sectional imaging data, coded as a set of stages that are co-expressed to a different extent in each individual. SuStaIn clusters individuals into groups (progression subtypes), based on the predominant expression of a given progression pattern. SuStaIn combines clustering and disease progression modelling to identify subgroups of individuals with distinct progression patterns. Disease progression modelling enables the reconstruction of disease progression patterns from cross-sectional data by modelling the expected properties of cross-sectional datasets given a particular progression pattern. For example, if Biomarker A becomes abnormal before Biomarker B, it would be expected that a proportion of individuals in a cross-sectional dataset have abnormal values for Biomarker A but normal values for Biomarker B. This property compares the relative likelihood of different candidate progression patterns. For detailed formalization and mathematical modelling of SuStaIn see previous publications.^27^ In Fig. 1, we provide a conceptual overview of the application of the SuStaIn algorithm in our study and dedicate the following paragraphs to briefly overview the methodology and outline parameter choices specific to our analyses. We used the SuStaIn algorithm separately for focal epilepsies and IGE. We also repeated the same analyses in the external validation cohort data.

We used SuStaIn with the piecewise linear *z*-score model of disease progression to estimate the most likely sequence with which selected regions reach different atrophy levels over time (Fig. 1B), i.e. to identify spatiotemporal patterns of progression of atrophy (subtype). Each subtype is described as a series of stages, whereby each stage corresponds to a biomarker (cortical thickness or volume of a brain region) reaching a new *z*-score. As previously reported, the optimal number of subtypes was determined using information criteria calculated through cross-validation^48^ to balance model complexity with internal model accuracy.^27^ Briefly, the piecewise linear *z*-score model requires *z*-scored data as input. Thus, each regional volume measurement was expressed as a *z*-score relative to the control group by normalizing each dataset relative to its control population in each institution, so that the control population had a mean of 0 and standard deviation (SD) of 1.^27^ As the UCL cohort consisted of participants acquired with two different scanners (3 T GE Signa, ‘old scanner’; and GE Discovery, ‘new scanner’; details above), people imaged with the old/new scanner were normalized to control subjects imaged with the old/new scanner only. In the context of progressive, disease-associated atrophy, regional cortical thinning and subcortical volumes decrease over time; thus, regional *z*-scores also become negative as a disease progresses. The piecewise linear *z*-score model, however, requires that *z*-scores increase as a function of disease progression. Hence, we multiplied the above-obtained *z*-score by −1 to allow for model fit, as previously described.^27^ We then ran the SuStaIn algorithm with 25 start points and 1 000 000 Markov Chain Monte Carlo (MCMC) iterations, as previously described,^27^ and evaluated solutions up to a maximum of *n* = 4 clusters (progression subtypes); the data-driven output of the SuStaIn algorithm, run separately in focal epilepsies and IGE, and the model fit and the choice of the number of clusters are discussed in the Supplementary material. We then performed 10-fold cross-validation (Fig. 1B) to evaluate the optimal number of clusters that best describe unseen data and assess the stability of progression subtypes across folds; the cross-validation similarity metric for the progression subtypes across validation folds ranges from 0 (no similarity) to 1 (maximum similarity).^27^

Importantly, each of the SuStaIn-identified progression subtypes is co-expressed to a different extent in each participant with epilepsy with values ranging from 0 to 1, so that their within-individual sum amounts to 1. For categorical classification purposes, we then assigned each individual with epilepsy to their primary progression subtype, based on the maximum likelihood of expression using a cut-off value of >50%, following prior work.^27,29^ Finally, we quantified the proportion of individuals classified into each (primary) subtype. SuStaIn also calculates the probability (maximum likelihood) with which each individual falls into a stage of each progression subtype. We staged individuals by computing their average SuStaIn stage, weighted by the probability that they belonged to each stage of each subtype.^27^ SuStaIn classifies individuals with no abnormalities in thickness or volume in any region as ‘weighted stage 0’ and they are not assigned to a progression subtype. In our study, there were no individuals assigned to weighted stage 0.

### Statistical analysis

Data were analysed using IBM SPSS version 26 and R 4.2.1. For demographic and clinical data, we used ANOVA, Kruskal-Wallis and chi-squared tests for continuous parametric, non-parametric and categorical data, respectively. To assess the relationship between SuStaIn stages and clinical characteristics, we employed two-tailed, non-parametric Spearman’s rank correlations with 95% confidence intervals (CI). These correlations were computed using 5000 bootstrapped random samples. In the discovery cohort, we also applied principal component analysis (PCA) to further our understanding of the clinical relevance of the identified progression subtypes. In detail, we entered FBTCS occurring in the year before MRI,^49,50^ seizure frequency, ASMs trialled over life, and epilepsy duration in a PCA for people with focal epilepsy. Generalized tonic-clonic seizure (GTCS) in the year before MRI, ASMs trialled over life, and epilepsy duration were entered in a PCA for people with IGE. Across all individuals, we then probed associations between expression of a progression subtype and weight of the extracted principal components, which represent superordinate markers of disease severity, using two-tailed non-parametric correlation analyses.

## Results

Our discovery cohort included 814 participants after data quality checks: (i) 503 with focal epilepsy [TLE: 328; frontal lobe epilepsy (FLE): 88; parietal lobe epilepsy (PLE): 27; occipital lobe epilepsy (OLE): 5; unclassified focal epilepsy: 55], of whom 418 with a lateralised seizure focus; (ii) 193 with IGE [juvenile myoclonic epilepsy (JME): 46; juvenile absence epilepsy (JAE): 43; IGE unclassified with GTCS, as primary seizure type: 104]; and (iii) 118 healthy control subjects. After quality checks, the external validation cohort included 254 participants: (i) 122 with focal epilepsy (TLE/FLE/posterior quadrant: 50/50/22; 115 with a lateralized seizure focus); (ii) 61 with JME; and (iii) 71 healthy control subjects. Demographic and clinical characteristics are provided in Table 1. There were no significant differences in age at seizure onset between people with focal epilepsy and those with IGE in the discovery and validation cohorts (two-tailed, two-sample *t-*tests, discovery/validation) [*t*(181/630) = −1.19/1.62, *P* = 0.236/0.106].

### SuStaIn identifies three focal epilepsy progression subtypes

**Figure 2:**
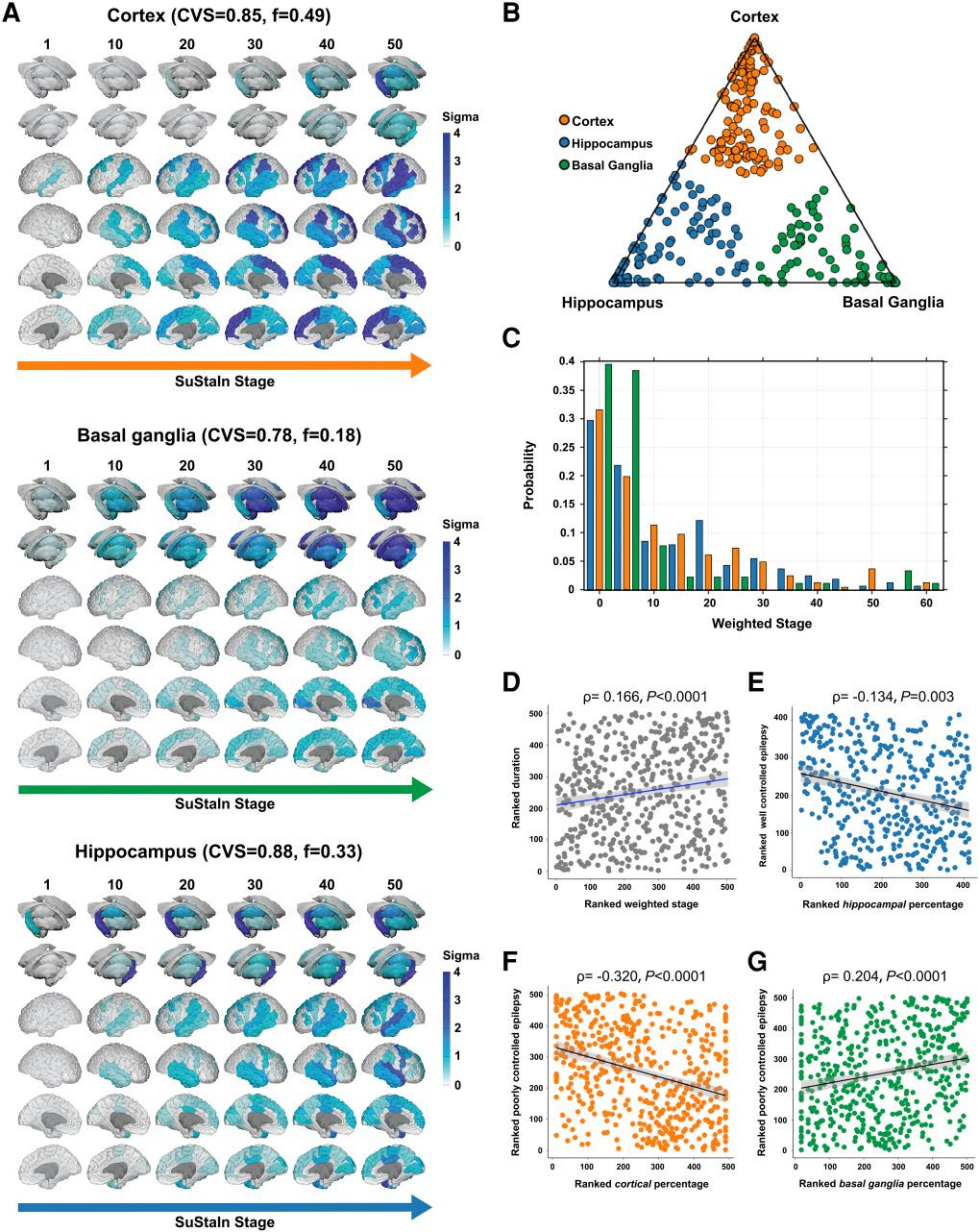
**MRI-based progression subtypes in focal epilepsy: discovery cohort.** The figure shows the spatiotemporal patterns of progression of grey matter atrophy (**A**: subtypes: cortical; basal ganglia; hippocampal) identified via SuStaIn in the focal epilepsy discovery cohort. Each of the three progression patterns in **A** consists of a sequence of stages with which cortical thickness and subcortical volumes reach different *z*-scores in people with epilepsy relative to healthy control subjects. The shading of each region indicates the severity of grey matter loss; white represents unaffected areas; light shading represents mildly affected areas (*z*-score = 1–2); medium shading represents moderately affected areas (*z*-score = 2–3); and dark shading represents severely affected areas (*z*-score >3). CVS = cross-validation similarity; *f* = proportion of participants assigned to each subtype. (**B**) The assignability of the disease subtype, operationalized as the distance from each vertex of the triangle, whereby each vertex represents the point at which membership of a given subtype is maximal (100%). Each participant was assigned to one subtype (cortical, basal ganglia or hippocampal) based on the maximum likelihood of subtype expression (cut-off value: > 50%). (**C**) The probability with which each participant from the focal epilepsy discovery cohort was assigned a specific SuStaIn stage (stage ranges: 0.002–62.424). (**D**) The correlation between duration of epilepsy and weighted stage. (**E**) A negative correlation is shown between within-individual expression of hippocampal subtype and a marker of well controlled epilepsy [principal component (PC2); see main text]. (**F** and **G**) Panels show the correlations between within-individual expression of cortical and basal ganglia subtypes and a marker of poorly controlled epilepsy (PC1). Correlation analyses were conducted with Spearman’s *ρ*; the associated panels show ranked data; Sigma (standard deviation), a measure of the spread of a dataset, is used to represent the variability of the data. SuStaIn = subtype and stage inference.

We identified three progression subtypes in focal epilepsy (Fig. 2A and B), each characterized by a sequence of stages (Fig. 2C): (i) a cortical progression subtype, dominant in 49.1% of cases (cross-validation folds: 0.85, 95% CI: 0.80–0.89) (Fig. 2A), characterized by atrophy initially encompassing the superior and transverse temporal gyri and parietal operculum, followed by the superior frontal, middle frontal and precentral cortices, then by the precuneus and posterior cingulate cortex, and by subcortical areas only in late stages; (ii) a basal ganglia subtype, dominant in 18.1% of cases (cross-validation folds: 0.78, 95% CI: 0.73–0.83) (Fig. 2A), with initial involvement of the globus pallidus, followed by other basal ganglia regions, thalamus and fronto-temporo-parietal cortices at later stages; and (iii) a hippocampal subtype, dominant in 32.8% of cases (cross-validation folds: 0.88, 95% CI: 0.83–0.92) (Fig. 2A), with a sequence first involving the hippocampus, followed by the thalamus, superior and middle temporal gyri, and then by other cortical areas.

**Figure 3:**
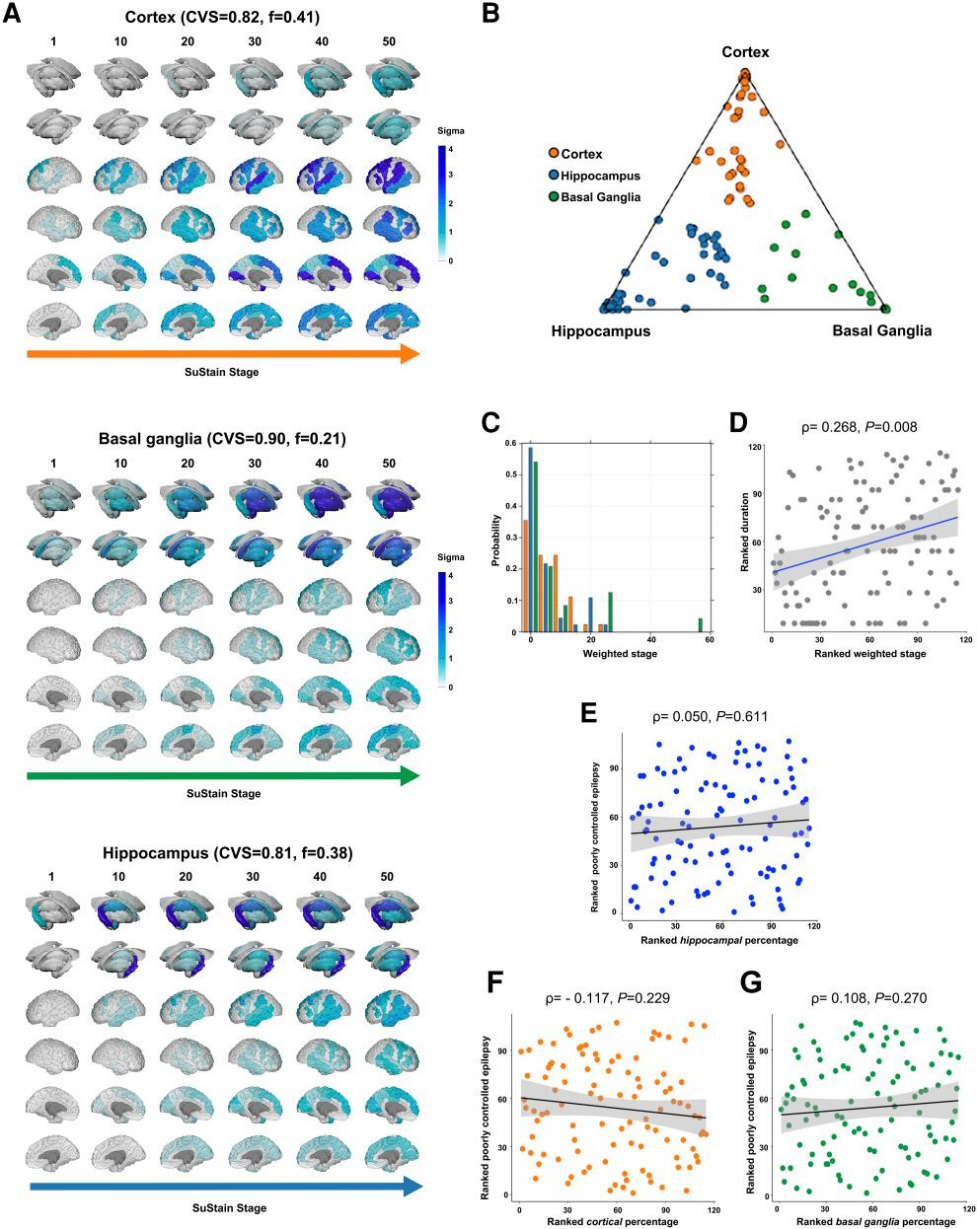
**MRI-based progression subtypes in focal epilepsy: validation cohort.** The figure shows the spatiotemporal patterns of progression of grey matter atrophy (**A**: subtypes: cortical; basal ganglia; hippocampal) identified via SuStaIn in the focal epilepsy validation cohort. Each of the three progression patterns in **A** consists of a sequence of stages with which cortical thickness and subcortical volumes reach different *z*-scores in patients relative to healthy control subjects. The shading of each region indicates the severity of grey matter loss; white represents unaffected areas; light shading represents mildly affected areas (*z*-score = 1–2); shading represents moderately affected areas (*z*-score = 2–3); and dark shading represents severely affected areas (*z*-score >3). CVS = cross-validation similarity; *f* = proportion of participants assigned to each subtype. (**B**) The assignability of the disease subtype, operationalized as the distance from each vertex of the triangle, whereby each vertex represents the point at which membership of a given subtype is maximal (100%). Each participant was assigned to one subtype (cortical, basal ganglia or hippocampal) based on the maximum likelihood of subtype expression (cut-off value: >50%). (**C**) The probability with which each participant from the focal epilepsy discovery cohort was assigned a specific SuStaIn stage (stage ranges: 0.006–54.008). (**D**) The correlation between duration of epilepsy and weighted stage (Spearman’s *ρ =* 0.268, *P* = 0.008), with an increasing weighted stage relating to longer disease duration; the associated panels show ranked data. (**E**–**G**) The not significantly important correlation is shown between a marker of poorly controlled epilepsy (PC1) with within-individual expression of hippocampal, cortical and basal ganglia subtypes. Correlation analyses were conducted with Spearman’s *ρ*; the associated panels show ranked data; Sigma (standard deviation), a measure of the spread of a dataset, is used to represent the variability of the data. SuStaIn = subtype and stage inference.

In the external validation cohort, we also identified three subtypes (Fig. 3A–C) with comparable progression patterns and proportion of people counted under each subtype: cortical: 41% (cross-validation folds: 0.82, 95% CI: 0.73–0.91) (Fig. 3A); basal ganglia: 21.3% (cross-validation folds: 0.90, 95% CI: 0.87–0.93) (Fig. 3A); and hippocampal: 37.7% (cross-validation folds: 0.81, 95% CI: 0.71–0.91) (Fig. 3A). There were no significant differences in subtype prevalence between discovery and validation cohorts (χ^2^_2_ = 3.779, *P* = 0.151), which corroborates the generalizability of our findings. Cross-validation analyses showed high reproducibility, with average similarity among cross-validation folds >78/>81% for each subtype in the discovery/validation cohorts. There were slight differences between discovery and validation cohorts: in the cortical subtype, the initial stages of the progression sequence were reversed, with temporal involvement following fronto-central involvement. For the basal ganglia subtype, the caudate was affected first, followed by the globus pallidus and the thalamus.

### Clinical characterization of the focal epilepsy subtypes

We next characterized each subtype from a clinical stand point (Supplementary Table 2). The hippocampal subtype mainly included people with TLE (78.2%) and had the highest proportion of mesial TLE with hippocampal sclerosis (TLE-HS; 31.7%) compared to the cortical (13.4%) and basal ganglia (14.3%) subtypes (χ^2^_2_ = 20.92, *P* < 0.0001) (Supplementary Table 2). Most people included in the hippocampal subtype (75.8%) had at least weekly seizures compared to 50.2% and 50.5% in the cortical and basal ganglia subtypes, respectively (χ^2^_2_ = 45.99, *P* < 0.0001). More people assigned to the basal ganglia subtype had FBTCS in the year before MRI (83.5%, versus 32.4% and 35.8% of cortical and hippocampal subtypes; χ^2^_2_ = 75.93, *P* < 0.0001). These findings were virtually identical in the validation cohort (χ^2^_2_ > 20.56, all *P* < 0.0001). Across progression subtypes, SuStaIn stages were correlated with the duration of epilepsy in the discovery (Spearman’s *ρ* = 0.166, CI = 0.079–0.253, *P* < 0.0001; Fig. 2D) and external validation cohort (*ρ* = 0.268, *P* = 0.008, CI = 0.120–0.405; Fig. 3D), with an increasing weighted stage relating to longer disease duration. We found no evidence for putative merging events between the three subtypes (Supplementary Fig. 2). Correlations of SuStaIn stages with age at onset (*ρ* = 0.036, *P* = 0.418) and seizure frequency (*ρ* = −0.013, *P* = 0.766) were not statistically significant; the association between SuStaIn stages and the occurrence of FBTCS in the discovery/validation cohorts was also not significant (Kruskal-Wallis *H* = 2.81/1.03*, P* = 0.094/0.311). Lastly, in people with a proven lateralized epileptic focus (*n* = 418/122, discovery/validation cohort), ipsilateral cortico-subcortical regions atrophied earlier than contralateral counterparts, irrespective of progression subtype (Supplementary Fig. 3). We performed additional analysis on the discovery focal epilepsy cohort, dividing it into two separate groups: individuals with TLE (*n* = 328) and those with extratemporal lobe epilepsy (*n* = 175). Three similar subtypes (cortical, basal ganglia, and hippocampal) were identified. We found significant differences between these cohorts in the origin of brain regions in the cortex-led subtype. Specifically, the TLE-only group showed earliest atrophy in temporal regions, while the extratemporal lobe epilepsy group displayed an origin of frontal regions (Supplementary Fig. 4).

In the discovery cohort, PCA on clinical characteristics (seizure frequency, disease duration, occurrence of FBTCS, and ASMs trialled over life) yielded two PCs with eigenvalues >1 (Supplementary material, ‘Results’ section): (i) PC1 (32.2% explained variance), with positive loadings of life-time trialled ASMs and seizure frequency, which we used as a surrogate marker for poorly controlled epilepsy; and (ii) PC2 (26.0% explained variance), with positive loading of epilepsy duration and negative loadings of FBTCS and seizure frequency, which we used as a marker of (chronic) well controlled epilepsy.

In the validation cohort, PCA with the same clinical characteristics yielded two PCs with eigenvalues >1 (Supplementary material, ‘Results’ section): (i) PC1 (38.0% explained variance), with positive loadings of illness duration, lifetime trialled ASMs and seizure frequency, which we used as a surrogate marker for poorly controlled epilepsy; and (ii) PC2 (25.9% explained variance), with negative loadings of life-time trialled ASMs and seizure frequency, which we used as a marker of well controlled epilepsy but none of these are correlated with the expression of three subtypes (*P* > 0.25).

Within-individual expression of the hippocampal subtype was associated with less expression of the well controlled epilepsy marker (*ρ* = −0.134, *P* = 0.003, CI = −0.216, −0.050; Fig. 2E). Expression of the cortical subtype was associated with less expression of the poorly controlled epilepsy marker (*ρ* = −0.320, *P* < 0.0001, CI = −0.397, −0.240; Fig. 2F), while the opposite relationship held true for the expression of the basal ganglia subtype (*ρ* = 0.204, *P* < 0.0001, CI = 0.120–0.285; Fig. 2G).

In the discovery cohort, 21.4% had epilepsy surgery, and in the validation cohort, 27.9%, with no significant differences among subtypes regarding the proportion of those having surgery (Supplementary Table 2). In the discovery cohort, people in the basal ganglia subtype had lower chances of a good postsurgical seizure outcome (50% Engel classes I–II) compared with those in the cortical (81.3%) and hippocampal (73.7%) subtypes (χ^2^_2_ = 7.41, *P* = 0.026); within-individual expression of the basal ganglia subtype was also negatively correlated with surgical outcome class (Spearman’s *ρ* = −0.238, *P* = 0.013). Findings in the validation cohort were qualitatively similar (good outcome: 83.3%, 82.3%, and 40% in cortical, hippocampal, and basal ganglia subtypes), but group differences (χ^2^_2_ = 4.337, *P* = 0.114) and correlation between within-individual basal ganglia subtype expression and surgical outcome class (Spearman’s *ρ* = −0.210, *P* = 0.303) were not statistically significant, likely owing to the small surgical sample (*n* = 34).

### SuStaIn identifies two idiopathic generalized epilepsy progression subtypes

**Figure 4:**
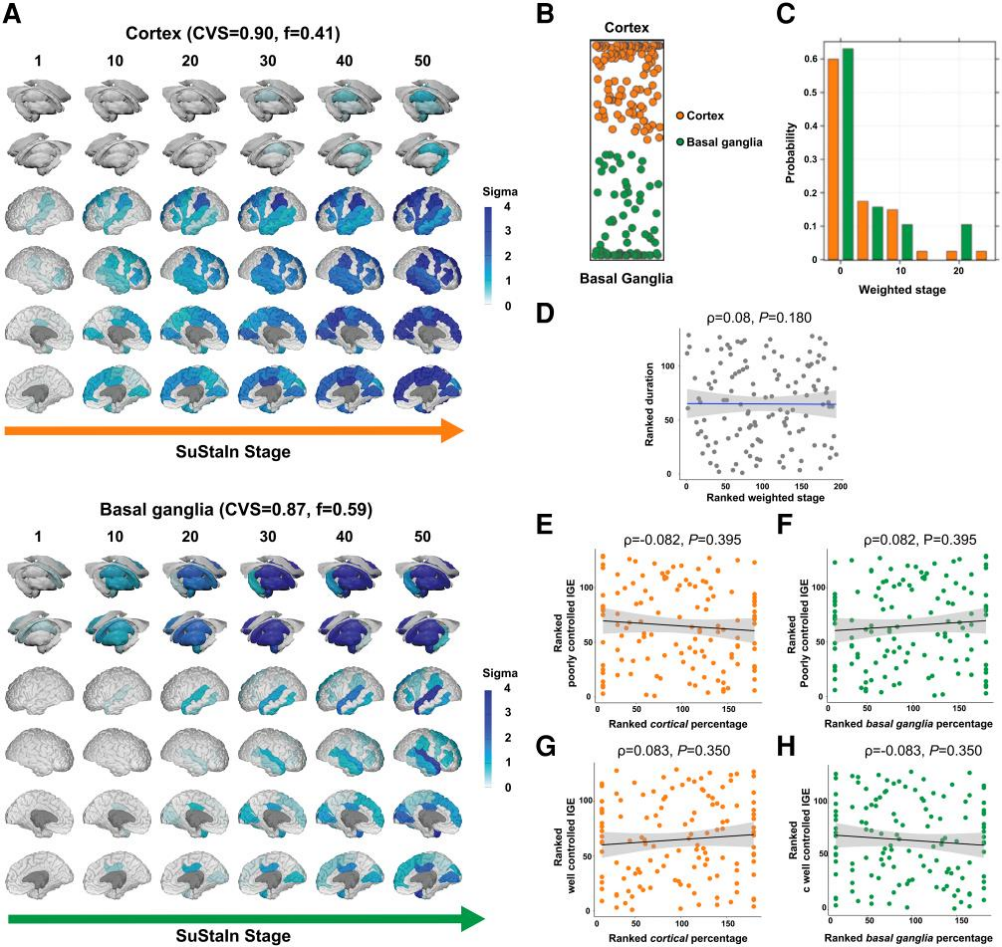
**MRI-based progression subtypes in IGE: discovery cohort.** The figure shows the spatiotemporal patterns of progression of grey matter atrophy (**A**: subtypes; cortical; basal ganglia) identified via SuStaIn in the IGE discovery cohort. (**A**) The colour of each region indicates the severity of grey matter loss; white represents unaffected areas; light shading represents mildly affected areas (*z*-score = 1–2); medium shading represents moderately affected areas (*z*-score = 2–3); and dark shading represents severely affected areas (*z*-score >3). CVS = cross-validation similarity; *f* = proportion of participants assigned to each subtype. (**B**) The assignability of the disease subtype, operationalized as the distance from each side of the bar, whereby each vertex represents the point at which membership of a given subtype is maximal (100%). (**C**) The probability with which each participant from the IGE discovery cohort was assigned a specific SuStaIn stage (stage ranges: 0.005–39.384). (**D**) The correlation between duration of epilepsy and weighted stage, which was not significant. (**E** and **F**) The correlations between within-individual expression of cortical and basal ganglia subtypes and a marker of poorly controlled IGE (PC1), which were not significant. (**G** and **H**) The correlations between within-individual expression of cortical and basal ganglia subtypes and a marker of well controlled IGE (PC2). Correlation analyses were conducted with Spearman’s *ρ*; the associated panels show ranked data; Sigma (standard deviation), a measure of the spread of a dataset, is used to represent the variability of the data. SuStaIn = subtype and stage inference.

**Figure 5:**
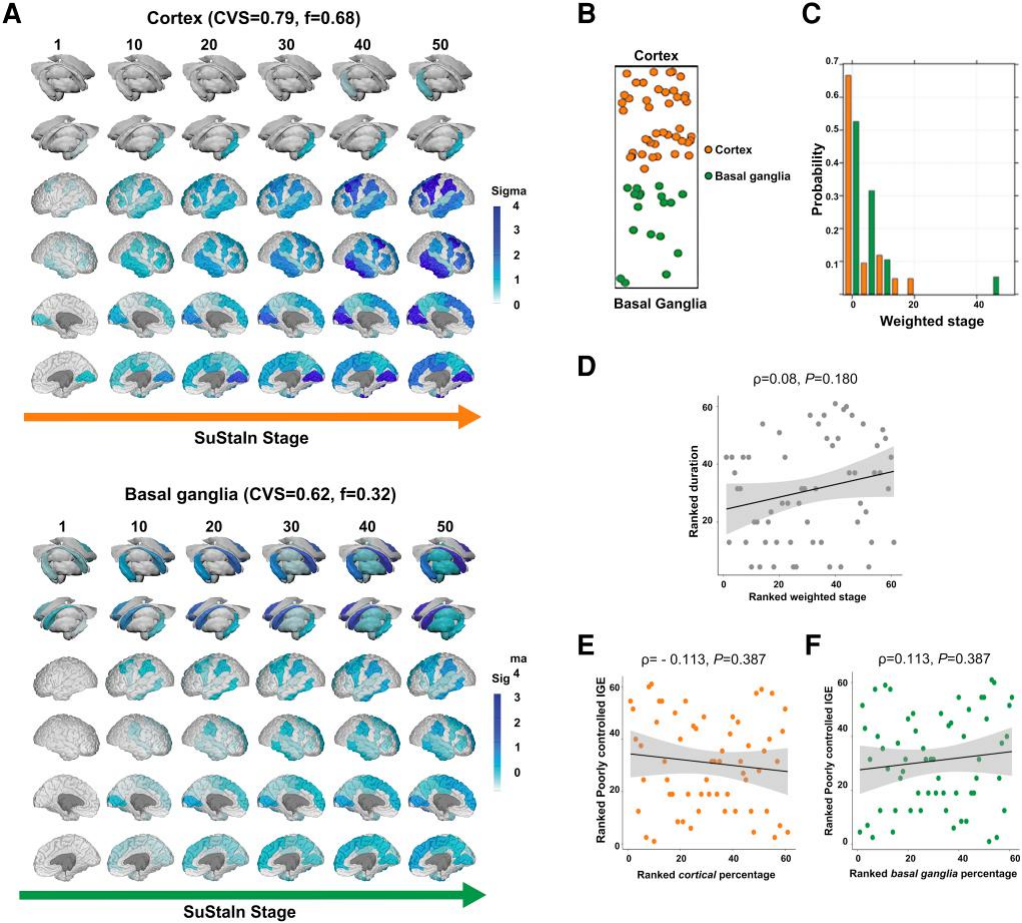
**MRI-based progression subtypes in IGE: validation cohort.** The figure shows the spatiotemporal patterns of progression of grey matter atrophy (**A**: subtypes; cortical; basal ganglia) identified via SuStaIn in the IGE validation cohort. (**A**) The shading of each region indicates the severity of grey matter loss; white represents unaffected areas; light shading represents mildly affected areas (*z*-score = 1–2); medium shading represents moderately affected areas (*z*-score = 2–3); and dark shading represents severely affected areas (*z*-score >3). CVS = cross-validation similarity. *f* = proportion of participants assigned to each subtype. (**B**) The assignability of the disease subtype, operationalized as the distance from each side of the bar, whereby each vertex represents the point at which membership of a given subtype is maximal (100%). (**C**) The probability with which each participant from the IGE discovery cohort was assigned a specific SuStaIn stage (stage ranges: 0.004–53.981). (**D**) The correlation between duration of epilepsy and weighted stage (Spearman’s *ρ*), which was not statistically significant; the associated panels show ranked data. (**E** and **F**) The (not significant) correlation between a marker of poorly controlled epilepsy (PC) with within-individual expression of cortical and basal ganglia subtypes. Correlation analyses were conducted with Spearman’s *ρ*; the associated panels show ranked data; Sigma (standard deviation), a measure of the spread of a dataset, is used to represent the variability of the data. IGE = idiopathic generalized epilepsy; SuStaIn = subtype and stage inference.

In IGE, SuStaIn yielded two progression subtypes, with largely overlapping findings in both cohorts (Fig. 4A, discovery cohort; Fig. 5A, validation cohort): (i) a cortical subtype, with 40.4% and 68.9% of people in the discovery and validation cohorts (cross-validation folds = 0.90, 95% CI = 0.82–0.90, discovery cohort, Fig. 4A; 0.79, 95% CI = 0.74–0.82, validation cohort, Fig. 5A); and (ii) a basal ganglia subtype, including 59.6% and 31.1% of people in the discovery and validation cohorts (cross-validation folds = 0.87, 95% CI = 0.80–0.88, discovery cohort, Fig. 4A; 0.62, 95% CI = 0.60–0.67, validation cohort, Fig. 5A). There were no differences in subtype distribution between the two cohorts (χ^2^_1_ = 1.37, *P* = 0.504). Spatiotemporal sequences of atrophy in both IGE subtypes were similar to those in focal epilepsy, with temporoparietal regions and the globus pallidus affected first in the cortical and basal ganglia IGE subtypes. To test the stability of two subtypes in IGE, we performed an additional analysis by combining people with focal epilepsy and IGE in the discovery cohort, which revealed that those with IGE are predominantly represented in the cortex-led and basal ganglia-led subtypes (Supplementary Fig. 5).

### Clinical characterization of the idiopathic generalized epilepsy subtypes

In the IGE discovery group, 66.7% of people assigned to the cortical subtype had absence or JME, while 67.8% of those assigned to the basal ganglia subtype had unclassified IGE with GTCS as primary seizure type (χ^2^_1_ = 26.19, *P* < 0.0001) (Supplementary Table 3). There were similar findings in the validation cohort: 73.7% of people with JME in the basal ganglia subtype had GTCS in the year before MRI, compared to 16.7% of those in the cortical subtype (χ^2^_1_ = 29.06, *P* < 0.0001) (Supplementary Table 3). In IGE, SuStaIn stages were not significantly associated with epilepsy duration (*ρ* = 0.028/0.08, *P* = 0.68/0.18; discovery/validation cohort) (Figs 4D and 5D) nor with age of onset (*ρ* = 0.036/−0.091, *P* = 0.684/0.486, discovery/validation cohort). In the discovery cohort, PCA on clinical characteristics (GTCS in the year before the scan, epilepsy duration, and ASMs trialled over life) generated two PCs with eigenvalues >1 (Supplementary material, ‘Results’ section): (i) PC1 (42.0% explained variance), with positive loadings of the number of life-time trialled ASMs and GTCS in the year before MRI, which we operationalized as a marker of poorly controlled IGE; and (ii) PC2 (34.2% explained variance), with positive and negative loading of the duration of epilepsy and GTCS, which we operationalized as a marker of well controlled IGE. There were no significant correlations between individual-level cortex or basal ganglia subtype expression and PC1 or PC2 (all *P* > 0.35) (Fig. 4E–H).

PCA with the same clinical characteristics in the validation cohort generated one PC eigenvalue >1: PC1 (40.8% explained variance), with positive loadings of illness duration and lifetime trialled ASMs, which we used as a surrogate marker for poorly controlled epilepsy, which are not correlated with expression of either subtype (*P* > 0.11).

## Discussion

We assessed 1105 people with epilepsy who underwent high resolution structural MRI in two specialist epilepsy centres. We used SuStaIn, an established machine learning algorithm that infers longitudinal sequences of progression of grey matter atrophy from cross-sectional data. Our findings indicate that focal epilepsy and IGE present with latent spatiotemporal patterns of progression, characterized by cortical or basal ganglia drivers of atrophy, that are differentially co-expressed in each individual. A subtype exclusive to focal epilepsy captured the progression of grey matter damage starting from the hippocampus. Analyses in the external validation cohort corroborated result generalizability. We also identified associations between progression subtypes and markers of disease severity and chronicity, supporting our findings’ clinical relevance. Our study provides dimensional evidence in a categorical framework. It delivers an innovative, imaging and AI-guided epilepsy taxonomy that may be leveraged for future advancements in individualized prognostics and targeted therapeutics.

Prior research into cumulative grey matter changes in epilepsy focused on proving its occurrence but did not address the temporal signatures of its unfolding. Our work conveys instead a comprehensive overview of spatiotemporal trajectories of progression. It establishes that these (i) imply a complex cortico-subcortical interplay; (ii) are simultaneously co-expressed to a different extent in each individual; and (iii) vary systematically among them. As the set of coordinated changes that underlie progression trajectories is individual-specific, it can thus be viewed as a spectrum e.g. as a dimensional entity. Identifying discrete progression subtypes, however, allows us to parse interindividual variability into categories, providing a compact classification framework that highlights the main patterns of vulnerability to atrophy. We classify patients by assigning them to categories to help us understand and have homogenous groups to study. However, it is important to acknowledge that in reality, there exists a biological continuum, such as the spectrum between focal and generalized seizures or the continuum between JAE and JME.

In addition, we note that previous work primarily assessed cumulative grey matter loss in people with focal epilepsy.^13,14,18^ Here we show that reorganization of brain structure over time also affects IGE. Our findings thus challenge prior views of IGEs as static disorders,^51^ and advocate for future research into strategies to mitigate progression other than surgery. More broadly, our application of SuStaIn favours a reconceptualization of the epilepsies as dynamic disorders associated with a deterioration of brain structure, indicating shared features with classical neurodegenerative disorders.^27,29,30,32^ These findings incentivize prompt diagnosis and treatment, and the search for disease-modifying therapy.^52^

Progression trajectories involving the cortex or the basal ganglia had broadly similar spatiotemporal characteristics in both focal and generalized epilepsies. Thus, despite their distinct clinical profiles, the substrates of neural vulnerability may be construed as trans-syndromic, and prompt a reassessment of the long-standing dichotomization between focal and generalized syndromes.^3^ In this context, we echo prior multicentre, cross-sectional evidence for common neuroanatomical signatures of epilepsy,^17,18^ and innovate by proving that these are also dynamic. The overlap in sequences of structural reorganization may suggest shared pathophysiology. As the epilepsies are increasingly conceptualized as network phenomena,^53^ it is plausible that the progression of structural damage may unfold across common large-scale, distributed neural networks, as observed in classical neurodegenerative disorders.^54^ Our work encourages a spectrum-based, dimensional conceptualization of the epilepsies that should complement a purely categorical view, echoing recent endeavours in modern psychiatry.^55,56^

As for the underlying circuitry, atrophy in the cortical subtypes first encompasses frontotemporal regions, then progresses to involve parieto-occipital areas, whose structural alterations were previously shown in cross-sectional work,^18,57^ and affects the hippocampus or subcortical regions only at late stages. The sequence of lateral temporal and frontocentral involvement differed between discovery and validation cohorts in focal epilepsies. Diagnostic characteristics may partly explain this finding: the discovery cohort had a predominance of people with TLE (∼60%) and a temporo-frontal sequence; in contrast, the validation cohort had people with TLE and FLE in equal proportion and a frontotemporal sequence. One possibility is that cortical areas near the epileptogenic networks may be affected first. This is supported by the finding that people with focal epilepsy had an earlier involvement of ipsilateral regions, irrespective of diagnostic group. In addition, such interpretation reiterates our previous findings in a longitudinal study^14^ and evidence of graded severity of diffusion abnormalities in TLE as a function of the Euclidean distance from the seizure focus.^58^ Future work may benefit from larger samples of people with extratemporal epilepsies and people with well established seizure focus lateralization and localization to disentangle their influence on progression. In IGE, progression trajectories in the cortical subtype broadly recapitulated those of focal epilepsies, with minor differences between the discovery and validation cohorts that may relate to heterogeneity in clinical characteristics. Half of the people in the discovery cohort were diagnosed with unclassified IGE, while people in the validation cohort had JME. Our findings in IGE implicate areas previously identified as atypical in cross-sectional imaging studies.^59-62^ Longitudinal studies investigating trajectories of structural reorganization in IGE are rare. In JME, altered development of association cortices occurs in the first 2 years after diagnosis, with an attenuated age-related decline in thickness and surface area compared to typical neurodevelopment.^63^ Such evidence was obtained in a paediatric cohort, while our IGE samples had an average age of >20 years. Future research is thus needed to characterize lifelong patterns of cortico-subcortical reorganization in IGE and compare trajectories in childhood and adolescence to those in adulthood.

We identified a basal ganglia progression subtype in focal and generalized epilepsies, which showed similar patterns of spatiotemporal evolution, first involving the globus pallidus, then the caudate and thalamus, followed by cortical areas. People with focal epilepsy and predominance of this subtype were more likely to have FBTCS, while those with IGE were more likely to be unclassified IGE with GTCS. Multimodal evidence from animal models and humans implicates thalamocortical and basal ganglia circuitry in generating or modulating tonic-clonic seizures.^16,49,64-68^ In IGE, imaging studies have documented subcortical grey matter volume loss, particularly in the thalamus,^16,69^ and reorganization of structural and functional thalamocortical connectivity.^19^ EEG-functional MRI studies also implicated the thalamus in generating generalized spike-wave discharges and absence of seizures, and showed hyperconnectivity among basal ganglia.^70^ In TLE, multimodal evidence points to thalamic atrophy and reorganization of thalamic and basal ganglia connectivity,^17,18,71-73^ which appears more marked in TLE with FBTCS.^49,50,64,74^ Collectively, our findings link with prior evidence on the role of the thalamus and basal ganglia in tonic-clonic seizures, pointing to substantial similarities in the pathophysiology of focal and generalized epilepsy. We postulate that the circuitry involved in tonic-clonic ictogenesis undergoes structural damage more precociously.

In focal epilepsies, we also identified a progression subtype characterized by initial involvement of the hippocampus, followed by the thalamus and temporal neocortex, and subsequently by other cortical areas. The spatial distribution of this progression pattern resembles that of areas exhibiting grey matter alterations and implicated in ictogenesis in TLE, particularly TLE-HS.^17,18^ Our findings mirror the results of single-centre longitudinal imaging studies^14,75,76^ and our meta-analysis,^13^ showing progressive thalamic and hippocampal atrophy and demonstrated that areas affected by the progression of atrophy^14^ were structurally connected to the hippocampus. We thus reiterate that seizure onset, propagation and progressive brain damage may be closely linked, with regions preferentially implicated in seizures deteriorating first. Notably, not all people with the predominant hippocampal progression subtype had TLE. The hippocampus may exhibit heightened vulnerability to developmental, hormonal and environmental factors, which is corroborated by its involvement in a broad spectrum of brain disorders.^77-79^ Studies in healthy adults identified the hippocampus as a component of a late-developing brain network with significant vulnerability to the effects of ageing and disease,^54^ and prior investigations of extratemporal epilepsy documented subtle structural hippocampal alterations, such as hippocampal malrotation.^80,81^ It is thus possible that repeated seizures, even if not mesiotemporal, may ultimately lead to pathological mesiotemporal reorganization, which could then propagate to other regions following the main axes of hippocampal structural connectivity.

Despite clear unilateral hippocampal sclerosis, only 50% of long-term study participants remained seizure-free after anterior mesial temporal lobe resection.^33^ Basal ganglia subtype in focal epilepsy had worse surgical outcomes than the hippocampal subtype, highlighting the clinical utility of SuStaIn for outcome prediction. Secondary mesial temporal sclerosis can occur in epilepsy syndromes other than TLE due to frequent and prolonged seizures.^82^ Our focal epilepsy cohort consists mainly of people with refractory epilepsy, suggesting a relatively common occurrence of secondary mesial temporal sclerosis. Consequently, disease progression in these individuals may deviate from the expected clinical syndrome, emphasizing the data-driven approach of SuStaIn to reconcile clinical syndromes with atrophy and disease progression patterns influenced by regions affected secondarily by epilepsy or seizures rather than initial seizure onset zones.

Correlation analyses contextualized the identified epilepsy progression subtypes from a clinical viewpoint. In focal epilepsies, the duration of disease, but not age at onset, was associated with subtype staging. While replicating prior cross-sectional evidence,^13,18^ our findings indicate that the progression of grey matter damage along the topographical axes captured by each subtype is time-dependent but may not be substantially influenced by the developmental stage at diagnosis. Seizure frequency was higher in those with predominant hippocampal progression. It is tempting to speculate that a higher seizure burden may more severely affect the hippocampus and lead to a progression cascade involving interconnected areas, per our prior considerations on hippocampal and network-level vulnerability to disease. Correlation analyses cannot establish causality, which need validation in future longitudinal studies. PCAs showed associations between epilepsy severity and progression subtypes, suggesting more aggressive disease in the cortical and basal ganglia subtypes. These findings show how SuStaIn may be used for clinical stratification, with pertinent implications. As subtype expression and their combination are quantifiable within individuals, higher cortical and basal ganglia loading findings have prognostic implications and may prompt accelerated treatment pathways. Similarly, people with focal epilepsy and predominant basal ganglia-led progression benefited less from epilepsy surgery, which may stem from a higher burden of secondary generalization, an established predictor of unfavourable post-surgical outcome^83-85^; the latter finding can also be translated to clinical decision-making. In IGE, people with uncontrolled GTCS were preferentially assigned to the basal ganglia subtype. Still, we did not otherwise identify significant correlations between clinical characteristics and subtypes and their stages. The underlying determinants of progression in IGE and focal epilepsies may differ, despite the considerable overlap in neuroanatomical signatures. IGE is characterized by a complex polygenic aetiology,^86,87^ which may be an essential driver of interindividual differences in the expression of progression subtypes and their associations with clinical phenotypes. These hypotheses will require validation in future imaging-genetics investigations.

A recent study using MRI utilized the SuStaIn method to investigate TLE.^88^ In contrast to the *z*-score SuStaIn method we used,^27^ they utilized the event-based SuStaIn approach.^30^ They exclusively focused on TLE-HS and confirmed a sequence of MRI changes that aligns with prior longitudinal findings.^13,14,76^ Interestingly, they also observed a correlation between the stage of their modelling and the duration of illness, which is similar to our findings in focal epilepsy.

Several lines of research, including neuroimaging, neuropathology, neuropsychology and network neuroscience, have contributed to proposing various taxonomies of epilepsy, particularly addressing neurobehavioral comorbidities.^89^ Our current study used structural imaging data to explore multiple brain atrophy trajectories. The SuStaIn method presents a unique opportunity to integrate various data sources, such as imaging, cognition and genetics, and our future work seeks to incorporate these diverse data sources to examine further aspects of disease progression patterns, such as the influence of genetic factors and implications on cognition.

A strength of our work is the inclusion of an external validation cohort, which strongly supports generalizability. SuStaIn is an open-source algorithm widely applied to multicentre cohorts of people with neurodegenerative disorders, it only requires cross-sectional datasets to detect multiple spatiotemporal trajectories and provides probabilistic and quantitative data information for individualized inference.^27,29,32^ Thus, we employed state-of-the-art, previously validated methods to maximize reproducibility and replicability. Cortical thickness, hippocampal and subcortical volumes can be reliably and non-invasively quantified using structural MRI and are validated morphometric markers of neuronal loss.^17,18^ One limitation is using a parcellation scheme that does not cover the whole brain. We note that selecting the regions we used was to maximize the trade-off between accuracy and computational complexity and was motivated by the findings of large-scale multicentre studies of the ENIGMA-Epilepsy consortium.^17,18^ The validation cohort size, especially in individuals with IGE, is relatively small. Longitudinal data on IGE are limited, particularly among the adult population. Notably, our analysis indicates a lack of correlation between the SuStaIn stages and epilepsy duration in individuals with IGE. Further longitudinal studies in the IGE cohort will be necessary to address this issue. A subset of individuals with focal epilepsy in our study underwent presurgical assessments. Unfortunately, only a relatively small proportion had undergone surgery at the time of the study. Thus, we seek to develop a predictive model for post-surgical outcomes based on SuStaIn outputs in larger post-surgical populations in the future.

In conclusion, we evaluated over a thousand people with epilepsies using an unsupervised machine learning algorithm and routinely acquired structural MRI scans. We describe patterns of spatiotemporal progression of grey matter atrophy. Progression subtypes principally implicate neocortical and basal ganglia drivers both in focal and generalized epilepsies, and limbic circuitry in focal epilepsy only. They are differentially co-expressed in each individual, and relate to clinical indicators of disease severity. Classification of people with epilepsy capitalizes on the maximally expressed progression subtype at the personal level, conveys a dimensional perspective into a categorical framework, and conceptually advances the extant categorical classification approaches. By providing an individual-level characterization of the underlying biology, we offer deliverables that can be used prospectively to enhance individualized prognostic and therapeutic considerations. It may aid clinical stratification for future clinical trials of disease-modifying agents.

## Supplementary Material

awad284_Supplementary_Data

## Data Availability

Anonymized statistical data to reproduce the main findings are available from the corresponding author upon reasonable request from any qualified investigator. Other data are not available due to their containing information that could compromise the privacy of research participants. Python and MATLAB implementations of the SuStaIn algorithm are available on the UCL-POND GitHub page: https://github.com/ucl-pond.
