# Supplementary material for: Identification of different MRI atrophy progression trajectories in epilepsy by subtype and stage inference
Source: Brain. 2023 Oct 9;146(11):4702–16. doi: 10.1093/brain/awad284 (PMC10629797; doi:10.1093/brain/awad284)
Supplement: awad284_Supplementary_Data [file awad284_supplementary_data.pdf]

Supplementary Material for

Identification of different MRI atrophy progression trajectories in epilepsy by Subtype and Stage Inference

Supplementary Methods

MRI data: quality control and participant exclusion

For MRI data of the discovery cohort, acquired at UCL, scan quality was guaranteed by UCL radiographers in terms of whole-brain coverage, absence of artefact and signal losses; the scans were inspected and clinically reported by multiple experienced neuroradiologists. Scans which were labelled as poor in quality either in the radiographers' notes or by the neuroradiologists were not considered for this study. For MRI data of the validation cohort, authors DA, WYL, and DZ carried out quality control checks for the scans of people with focal epilepsy, while DA and YZ carried out those for the scans of people with JME. All individuals with scans of insufficient quality were not considered for this study. Before MRI data preprocessing, a total of 894 people with epilepsy [691 with focal epilepsy (336/355 old/new UCL scanner) and 203 IGE (all old scanner)] and 121 healthy control participants (50/71 acquired with the old/new UCL scanner) from the discovery cohort, and 211 people with epilepsy (150 with focal epilepsy/61 with IGE) and 73 control participants from the validation cohort, for a total of 1299 scans, were considered for inclusion in this work.

The Computational Anatomy Toolbox (CAT) introduces a quality control framework and allows evaluating essential image parameters such as noise, signal inhomogeneities, and image resolution. All these quality measures are subsequently used in the context of a rating scale system that enables the comparison of measures across different scanners and sequences. Moreover, quality measures are summarised into a single quality rating with the following possible options: excellent, good, satisfactory, sufficient, critical, and failed. Only scans rated "excellent" or "good" were considered for inclusion and visually inspected, while those not reaching these quality thresholds were discarded. In the discovery cohort, 540 scans of people with focal epilepsy and 195 with IGE were rated as "excellent" or "good", while 151 scans of people with focal epilepsy and 10 scans of people with IGE (all acquired with the old UCL scanner) had inferior quality and were excluded; moreover, an additional 37 scans of people

with focal epilepsy, that were rated as "good" but were quantitatively on the low side of the CAT12 "good" rating spectrum, were discarded after visual inspection. The scans of 3 controls (2 old scanner/1 new scanner) were also excluded for low quality ratings. Thus, the final participant number of the discovery cohort consisted of 503 people with focal epilepsy, 193 with IGE, and 118 healthy control participants. In the validation cohort, 122 scans of people with focal epilepsy, 61 (all) scans of people with IGE and 71 of 73 control scans were rated as "excellent" or "good", while 28 scans of people with focal epilepsy and 2 control scans were excluded for lower quality ratings. Thus, the final participant number of the validation cohort consisted of 122 people with focal epilepsy, 61 people with IGE, and 71 healthy controls.

Supplementary Results

Principal Component Analysis

These analyses were conducted on discovery cohort data. To further assess the clinical relevance of MRI-based progression subtypes, we first derived composite clinical constructs by entering clinical characteristics into a principal component analysis (PCA), and then assessed the relationship between the former and within-individual subtype expression using Spearman's rank correlations. In focal epilepsy, a PCA based on seizure frequency, disease duration, the occurrence of FBTCS (entered as binary variable: yes/no), and antiseizure medications (ASMs) trialled over life yielded two principal components (PCs) with eigenvalues >1: (i) PC1 (eigenvalue=1.29, 32.2% of explained total variance), with positive loadings of lifetime trialled ASM (0.826) and seizure frequency (0.584), which we operationalized as a superordinate marker of poorly controlled (i.e., chronic and active) epilepsy; and (ii) PC2 (eigenvalue=1.04, 26.0% of explained total variance), with positive loading of epilepsy duration (0.797) and negative loading of FBTCS (-0.613) and seizure frequency (-0.169), which we operationalized as a superordinate marker of (chronic) well controlled epilepsy. In IGE, a PCA on disease duration, occurrence of GTCS in the year before MRI, ASMs trialled over life yielded two PCs with eigenvalues >1: (i) PC1 (eigenvalue=1.19, 42.0% of explained total variance), with positive loading of number of lifetime trialled ASMs (0.645) and GTCS in the year before MRI (0.505), which we operationalized as a superordinate marker of poorly controlled IGE; (ii) and PC2 (eigenvalue=1.03, 34.2% of explained total variance), with a positive loading of duration of epilepsy (0.798) and negative loading of GTCS (-0.58), which we operationalized as a marker of chronic well controlled IGE. Statistical details

regarding correlation analyses are provided in the main manuscript text; the associated correlation scatterplots are displayed in Fig. 2 and Fig. 5.

Similarity between subtype progression patterns

We measured the similarity of progression patterns between two cohorts using the Bhattacharyya coefficient. Specifically, we evaluate the Bhattacharyya coefficient between the positional variance diagram, which are matrices converted from each biomarker event from progression patterns from each cohort, averaged across biomarker events and Markov Chain Monte Carlo samples. The Bhattacharyya coefficient is a metric that quantifies the similarity of the distribution of the position of biomarker events in the subtype sequences, ranging from 0 (maximum dissimilarity) to 1 (maximum similarity). A Bhattacharyya coefficient greater than 0.50 indicates a moderate to strong similarity between two probability distributions. It suggests that there is a significant overlap or resemblance between the distributions being compared.

In the context of our study on focal epilepsy and IGE, we calculated the Bhattacharyya coefficients between the discovery and validation cohorts (focal epilepsy-discovery v.s focal epilepsy validation; IGE-discovery v.s IGE-validation). Our results showed that the Bhattacharyya coefficients were 0.69 (>0.50) between focal epilepsy-discovery and focal epilepsy-validation cohorst and 0.58 (>0.50) between IGE-discovery and IGE-validation cohorts.

Bhattacharyya, A. K. On a measure of divergence between two statistical populations defined by their probability distributions. Sankhya Indian J. Stat.7, 401–406 (1943).

Model fit discussion

SuStaIn has been specifically designed to identify and differentiate subtypes or spectrums of subtypes. The algorithm optimises a data likelihood term, which can be utilised for model comparison within a given dataset. We used the exact some model/method in the reference *Young et al., Uncovering the heterogeneity and temporal complexity of neurodegenerative diseases with Subtype and Stage Inference, Nat Comm 2018*.

The ability of SuStaIn to recover the correct number of subtypes was assessed in the reference *Young et al., Nat Comm 2018* using simulated data with settings designed to be representative of the GENFI dataset (N=313, http://www.genfi.org.uk/) and ADNI (http://adni.loni. usc.edu) 3T (N=793) and 1.5T (N=576) datasets. Three datasets were simulated for each parameter setting and SuStaIn estimated the number of subtypes by implementing the full 10-fold cross-validation procedure and comparing the cross-validation information criteria for different subtype numbers. A set of default model parameters were tested and each parameter was varied individually to establish the effect of different settings on the recovery of the subtypes and stages by the SuStaIn algorithm: (i) the number of subjects J as $J = 200$, $J = 500$, and $J = 1000$; (ii) the number of biomarkers I as $I = 5$, $I = 10$, and $I = 15$; (iii) the number of clusters C as $C = 1$, $C = 3$, and $C = 5$.

Kendall rank correlations between the estimated subtype progression patterns and the ground truth for different numbers of subjects, increasing numbers of clusters and increasing number of the predetermined biomarkers were calculated. The distribution of the error in the proportion of subjects assigned to each cluster for different numbers of subjects was calculated for different subject numbers, biomarker numbers and cluster numbers. The distribution of the position of each event stage in the subtype progression patterns represents the uncertainty in the subtype progression patterns estimated by SuStaIn.

To ensure a good model fit, we considered the following points:

1. *Number of subjects*

   From the ref. *Young et al., Nat Comm 2018,* the distribution of the Kendall rank correlation between the estimated subtype progression patterns and the ground truth for different numbers of subjects: 200 subjects (comparable to the GENFI dataset), 500

subjects (comparable to the ADNI dataset), and 1000 subjects (comparable to the combination of the ADNI 3T and 1.5T). As expected, larger subject numbers correspond to increasing accuracy of the subtype progression patterns: the average Kendall rank correlation across the 10 simulated datasets is 0.86 for 200 subjects, 0.92 for 500 subjects, and 0.95 for 1000 subjects. However, even with only 200 subjects, there is a high enough Kendall rank correlation to provide a meaningful indication of the subtype progression patterns. For the distribution of the error in the proportion of subjects assigned to each cluster for different numbers of subjects, SuStaIn accurately estimates the proportion of individuals belonging to each cluster: the mean absolute error is 0.054 for 200 subjects (an expectation of about 10/200 subjects misassigned), 0.026 for 500 subjects, and 0.022 for 1000 subjects. For the uncertainty in the proportion of individuals for different numbers of subjects estimated by SuStaIn, this uncertainty decreases as the number of subjects increases, with an average standard deviation across experiments of 0.048 for 200 subjects, 0.027 for 500 subjects, and 0.019 for 1000 subjects.

We included 503 for focal epilepsy and 193 for IGE in the discovery cohort. Although there are less subjects (122 focal epilepsy; 61 IGE) in the validation cohort, it replicated the three subtypes in focal epilepsy and two subtypes in IGE.

2. *Number of biomarkers*

In the ref. *Young et al., Nat Comm 2018*, as the number of biomarkers increases, the accuracy of the subtype progression patterns stays more or less constant, with an average Kendall rank correlation of 0.90 for 5 biomarkers, 0.92 for 10 biomarkers, and 0.91 for 15 biomarkers. For the distribution of the error in the proportion of subjects assigned to each cluster for different numbers of biomarkers, SuStaIn accurately estimates the proportion of individuals belonging to each cluster: the mean absolute error is 0.053 for 5 biomarkers, 0.026 for 10 biomarkers, and 0.026 for 15 biomarkers. The uncertainty in the subtype progression patterns is larger for smaller numbers of biomarkers: the uncertainty in the position of each biomarker event has an average standard deviation of 0.09 for 5 biomarkers, 0.07 for 10 biomarkers, and 0.06 for 15 biomarkers. This is likely because the trajectories of different subgroups are more strongly defined and separated by larger numbers of biomarkers.

We have used 40 biomarkers, thus the mean absolute error is likely even less than the values stated above.

3. Number of clusters

As described in the ref. *Young et al., Nat Comm 2018*, the average Kendall rank correlation is 0.97 for 1 cluster, 0.92 for 3 clusters and 0.87 for 5 clusters. The distribution of the position of each event stage in the subtype progression patterns, i.e. the uncertainty in the subtype progression patterns estimated by SuStaIn, for different numbers of clusters. As expected, the uncertainty increases as the number of clusters increases, with an average standard deviation across simulated datasets of 0.03 for 1 cluster, 0.07 for 3 clusters, and 0.10 for 5 clusters. The uncertainty in the subtype progression pattern is largest for the least dominant cluster, which has the smallest number of subjects. The distribution of the error in the proportion of subjects assigned to each cluster for different numbers of clusters: the mean absolute error is 0 for 1 cluster (the fraction is always 1 for 1 cluster), 0.026 for 3 clusters, and 0.030 for 5 clusters. The distribution of the proportion of individuals belonging to each cluster, i.e. the uncertainty in the proportion of individuals estimated by SuStaIn, for different numbers of clusters. As expected, this uncertainty increases as the number of clusters increases, with an average standard deviation across experiments of 0 for 1 cluster, 0.027 for 3 clusters, and 0.027 for 5 clusters. The distribution is centred around zero, i.e. the estimate of the proportion of individuals belonging to each cluster is unbiased for varying numbers of clusters.

For varying numbers of clusters SuStaIn estimates the correct number of clusters in all simulated datasets for 1, 3 and 5 clusters.

We used 4 clusters (C=4) for focal epilepsy, which identify the same number of subtypes (3) when using 3 clusters (C=3), and 3 clusters (C=3) for IGE, identifying 2 subtypes

4. External validation

We validated progression patterns identified in the discovery cohort by repeating the analysis in an external validation cohort. Here, we identified very similar progression

patterns. Notably, a greater number of cases in the external cohort were found to be in the early stages compared to the discovery cohort, which was reflected in the difference in the illness duration between the two cohorts (9.9 years vs. 19.9 years). We could demonstrate that subjects within the cohort spanned various stages and subtypes, as evidenced by the histograms of the SuStaIn stage distribution for each subtype (Figure 2E, Figure 4), and in the external validation of progression patterns (Figure 3E).

Supplementary Table 1. Regions of Interest.

ROI types	***Brain areas***	***P value***	***Cohen's d***	***Preprocessing***
Cortical thickness (Desikan-Killiany Atlas, DK40)	Left caudal middle frontal	<0.001	-0.262	Regional cortical thickness values were computed after correction for age and sex and were transformed to *z*-scored relative to a control population. For the UCL discovery cohort, patient data acquired via the old/new scanner was *z*-scored based on controls acquired with the old/new scanner, respectively.
	Right caudal middle frontal	0.042	-0.073	
	Left cuneus	0.034	-0.255	
	Right cuneus	0.051	-0.167	
	Left inferior temporal	0.210	-0.270	
	Right inferior temporal	0.021	-0.420	
	Left lingual	0.536	-0.321	
	Right lingual	0.341	-0.368	
	Left middle temporal	0.009	-0.360	
	Right middle temporal	0.006	-0.588	
	Left paracentral	<0.001	-0.437	
	Right paracentral	0.090	-0.374	
	Left pars triangularis	0.056	-0.247	
	Right pars triangularis	0.149	-0.175	
	Left precentral	<0.001	-0.476	
	Right precentral	0.003	-0.335	
	Left precuneus	0.012	-0.049	
	Right precuneus	0.036	-0.206	
	Left posterior cingulate	0.087	-0.272	
	Right posterior cingulate	0.319	-0.301	
	Left superior frontal	0.017	-0.419	
	Right superior frontal	0.017	-0.170	
	Left superior temporal	0.002	-0.735	
	Right superior temporal	<0.001	-0.762	
	Left supramarginal	0.003	-0.616	
	Right supramarginal	0.002	-0.371	
	Left transverse temporal	0.176	-0.551	

	Right transverse temporal	0.067	-0.536	
Hippocampal volume (Hipposeg) and subcortical volumes (Geodesic Information Flow)	Left hippocampus	<0.001	-0.967	Regional volumetric values were computed after correction for age, sex, and total intracranial volume (TIV; head size correction purposes) and were transformed to *z*-scored relative to a control population. For the UCL discovery cohort, patient data acquired via the old/new scanner was *z*-scored based on controls acquired with the old/new scanner, respectively.
	Right hippocampus	<0.001	-0.755	
	Left amygdala	0.130	-0.045	
	Right amygdala	0.180	-0.039	
	Left caudate	0.040	-0.214	
	Right caudate	0.018	-0.119	
	Left pallidum	0.140	-0.668	
	Right pallidum	0.079	-0.606	
	Left putamen	0.339	-0.388	
	Right putamen	0.403	-0.327	
	Left thalamus	0.005	-0.588	
	Right thalamus	0.017	-0.534	

Supplementary Table 2. Characterisation of MRI-based focal epilepsy subtypes in the discovery and validation cohorts.

Discovery cohort				
	Cortex	*Basal ganglia*	*Hippocampus*	***P value*** *(For intergroup differences within cohort)*
Number of patients *(% of total sample)*	247 (49.1%)	91 (18.1%)	165 (32.8%)	N/A
Sex *(F/M)*	123/124	43/38	82/83	0.911
Age *mean (SD)*	35.7 (10.6)	33.7 (9.1)	35.6 (11.8)	0.264
Age of onset *mean (SD)*	16.0 (10.9)	14.9 (9.3)	15.0 (13.0)	0.579
Duration of epilepsy *mean (SD)*	19.7 (12.7)	18.7 (11.9)	20.7 (13.0)	0.473
Lateralization of seizure focus *(L/R/B/U, n)*	116/92/18/21	31/46/7/7	78/55/19/13	0.135
Localization of seizure focus *(n)*	TLE (143)/ FLE (48)/ PLE (19)/ OLE (4)/ U (33)	TLE (56)/ FLE (20)/ OLE (1) / U (14)	TLE (129)/ FLE (20)/ PLE (8)/ U (8)	0.504
Localization of seizure focus of cases with proven lateralisation *(n)*	TLE (130)/ FLE (43)/ PLE (17)/ OLE (4)/ U (14)	TLE (49)/ FLE (19)/ OLE (1)/ U (8)	TLE (101)/ FLE (18)/ PLE (7)/ U (7)	**0.030**
FBTCS† *(% of patients)*	80 (32.4%)	76 (83.5%)	59 (35.8%)	**<0.001**
HS *n (% of patients)*	33 (13.4%)	13 (14.3%)	52 (31.5%)	**<0.001**
Frequent seizures *> weekly; n (%)*	124 (50.2%)	46 (50.5%)	125 (75.8%)	**<0.001**
ASM *median (range)**	2 (0-6)	2 (0-5)	2 (0-5)	0.188
Surgery *n (% of patients) (Localization, n)*	48 (19.4%) (37 TLE/ 8 FLE/ 3 PLE)	22 (24.2%) (17 TLE/ 5 FLE)	38 (23.0%) (31 TLE/ 7 FLE)	0.538
Engel I-II surgical outcome *n (% of patients undergone surgery)*	39 (81.3%)	11 (50%)	28 (73.7%)	**0.026**
Post-operative follow-up *y, median (range)*	7.0 (3-14)	5.5 (2-15)	4.8 (3-14)	0.217
**Sustain Stage** *mean (SD)*	12.5 (14.5)	8.8 (15.3)	12.7 (13.7)	0.072
**Validation cohort**				
	Cortex	*Basal ganglia*	*Hippocampus*	***P value*** *(For intergroup differences within cohort)*
Number of patients *(% of total sample)*	50 (41.0%)	26 (21.3%)	46 (37.7%)	N/A
Sex *(F/M)*	20/30	9/17	20/26	**<0.001**
Age *mean (SD)*	25.8 (7.6)	23.4 (11.2)	25.1 (8.5)	0.561
Age of onset *mean (SD)*	17.2 (9.4)	15.5 (7.4)	16.8 (13.2)	0.682

Duration of epilepsy *mean (SD)*	8.9 (6.1)	6.6 (5.1)	9.7 (5.9)	0.112
Lateralization of seizure focus *(L/R/B/U, n)*	31/16/2/1	16/8/1/1	37/9/0/0	0.285
Localization of seizure focus *(n)*	TLE (16)/ FLE (24)/ PO (10)	TLE (3)/ FLE (17)/ PO (6)	TLE (31)/ FLE (9)/ PO (6)	**<0.001**
Localization of seizure focus of cases with proven lateralisation *(n)*	TLE (16)/ FLE (21)/ PO (10)	TLE (3)/ FLE (15)/PO (6)	TLE (31)/ FLE (9)/ PO (6)	**<0.001**
FBTCS† *(% of patients)*	16 (32.0%)	23 (88.5%)	22 (47.8%)	**<0.001**
HS *n (% of patients)*	9 (18.0%)	0	27 (58.7%)	**<0.001**
Frequent seizures *> weekly; n (%)*	26 (52%)	13 (50%)	42 (91.3%)	**<0.001**
ASM *median (range)**	2 (1-3)	2 (1-3)	2 (1-3)	0.910
Surgery *n (% of patients) (Localization, n)*	12 (24%) (10 TLE/ 2 PO)	5 (19.2%) (3 TLE/ 2 PO)	17 (37.0%) (16 TLE/ 1 PO)	0.199
Engel I-II surgical outcome *n (% of patients undergone surgery)*	10 (83.3%)	2 (40%)	14 (82.4%)	0.114
Post-operative follow-up *y, median (range)*	5.0 (4-7)	5.0 (4-5)	5.0 (3-7)	0.594
**Sustain Stage** *mean (SD)*	6.7 (6.3)	7.9 (13.2)	5.8 (7.1)	0.650

† Refers to at least one FBTCS experienced in the year prior to the investigation; * Kruskal Wallis Test. ASM: anti-seizure medications FBTCS: focal to bilateral tonic-clonic seizure(s); F: female; FLE: frontal lobe epilepsy; HC: healthy control; HS: hippocampal sclerosis; L: left; M: male; n: number; OLE: occipital lobe epilepsy; PLE: parietal lobe epilepsy; R: right; SD: standard deviation; TLE: temporal lobe epilepsy; U: unknown.

Supplementary Table 3. Characterisation of MRI-based IGE subtypes in the discovery and validation cohorts.

**Discovery cohort**			
	Cortex	***Basal ganglia***	***P value*** *(For intergroup differences within cohort)*
Percentage of total sample	78 (40.4%)	115 (59.6%)	N/A
Sex *(F/M)*	49/29	65/50	0.600
Age *mean (SD)*	30.8 (8.9)	35.7 (12.6)	0.018
Age of onset *mean (SD)*	13.7 (5.0)	14.1 (6.8)	0.840
Duration of epilepsy *mean (SD)*	22.1 (13.1)	18.1 (10.5)	0.059
Syndrome *(n)*	JAE (27)/ JME (25)/ GTCS-unc (26)	JAE (16)/ JME (21)/ GTCS-unc (78)	**<0.001**
GTCS† *(% of patients)*	26 (33.3%)	78 (67.8%)	**<0.001**
Frequent seizures *> weekly; n (%)*	43 (55.1%)	52 (45.2%)	0.400
ASM *median (range)**	2 (0-4)	2 (0-4)	0.833
**Sustain Stage** *mean (SD)*	3.2 (4.2)	7.0 (8.3)	**<0.001**
**Validation cohort**			
	Cortex	***Basal ganglia***	***P value*** *(For intergroup differences within cohort)*
Percentage of total sample	42 (68.9%)	19 (31.1%)	N/A
Sex *(F/M)*	15/27	12/7	**<0.001**
Age *mean (SD)*	19.5 (4.4)	22.5 (7.1)	0.472
Age of onset *mean (SD)*	13.3 (2.7)	14.0 (2.6)	0.936
Duration of epilepsy *mean (SD)*	6.1 (4.8)	8.3 (7.4)	0.211
Syndrome *(n)*	JME (42)	JME (19)	N/A
GTCS† *(% of patients)*	7 (16.7%)	14 (73.7%)	**<0.001**
Frequent seizures *> weekly; n (%)*	21 (50.0%)	15 (78.9%)	0.033
ASM *median (range)**	1 (0-2)	1 (0-2)	0.461
**Sustain Stage** *mean (SD)*	4.6 (9.3)	4.8 (9.8)	0.962

† Refers to at least one one GTCS experienced in the year prior to the investigation; ***** Kruskal Wallis Test. ASM: anti-seizure medications; GTCS: generalised tonic clonic

seizure; F: female; HC: healthy control; GTCS: generalized tonic-clonic seizure(s); GTCS-unc: IGE unclassified, with GTCS as main seizure subtype; IGE: idiopathic generalized epilepsy; JAE: juvenile absence epilepsy; JME: juvenile myoclonic epilepsy; L: left; M: male; n: number; R: right; SD: standard deviation;

Supplementary Table 4. Correlation analysis in the two cohorts

	Focal epilepsy-Discovery (Spearman ρ, 95% CI, p-value)	Focal epilepsyValidation (Spearman ρ, 95% CI, p-value)	IGE-Discovery (Spearman ρ, 95% CI, p-value)	IGE-Validation (Spearman ρ, 95% CI, p-value)
Expression of the hippocampal subtype with PC1 (poorly controlled epilepsy marker)	**0.057, n/a, 0.178**	**0.050, n/a, 0.611**	**n/a**	**n/a**
Expression of the hippocampal subtype with PC2 (well controlled epilepsy marker)	**-0.134, -0.216 - -0.050, 0.003**	**0.083, n/a, 0.395**	**n/a**	**n/a**
Expression of the cortical subtype with PC1 (poorly controlled epilepsy marker)	**-0.320, -0.397 - -0.240, <0.0001**	**-0.117, n/a, 0.229**	**-0.082, n/a, 0.395**	**-0.113, n/a, 0.387**
Expression of the cortical subtype with PC2 (well controlled epilepsy marker)	**-0.03, n/a, 0.938**	**-0.114, n/a, 0.242**	**0.083, n/a, 0.350**	**No PC2 generated in PCA analysis**
Expression of the basal ganglia subtype with PC1 (poorly controlled epilepsy marker)	**0.204, 0.120-0.285, <0.0001**	**0.108, n/a, 0.270**	**0.082, n/a, 0.395**	**0.113, n/a, 0.387**
Expression of the basal ganglia subtype with PC2 (well controlled epilepsy marker)	**0.017, n/a, 0.694**	**0.086, n/a, 0.378**	**-0.083, n/a, 0.350**	**No PC2 generated in PCA analysis**
Illness duration with weighted stage	**0.166, 0.079-0.253, <0.0001**	**0.268, 0.120-0.405, 0.008**	**0.028, n/a, 0.680**	**0.080, n/a, 0.180**

Supplementary Figure 1.
Cortical and subcortical regions of interest.

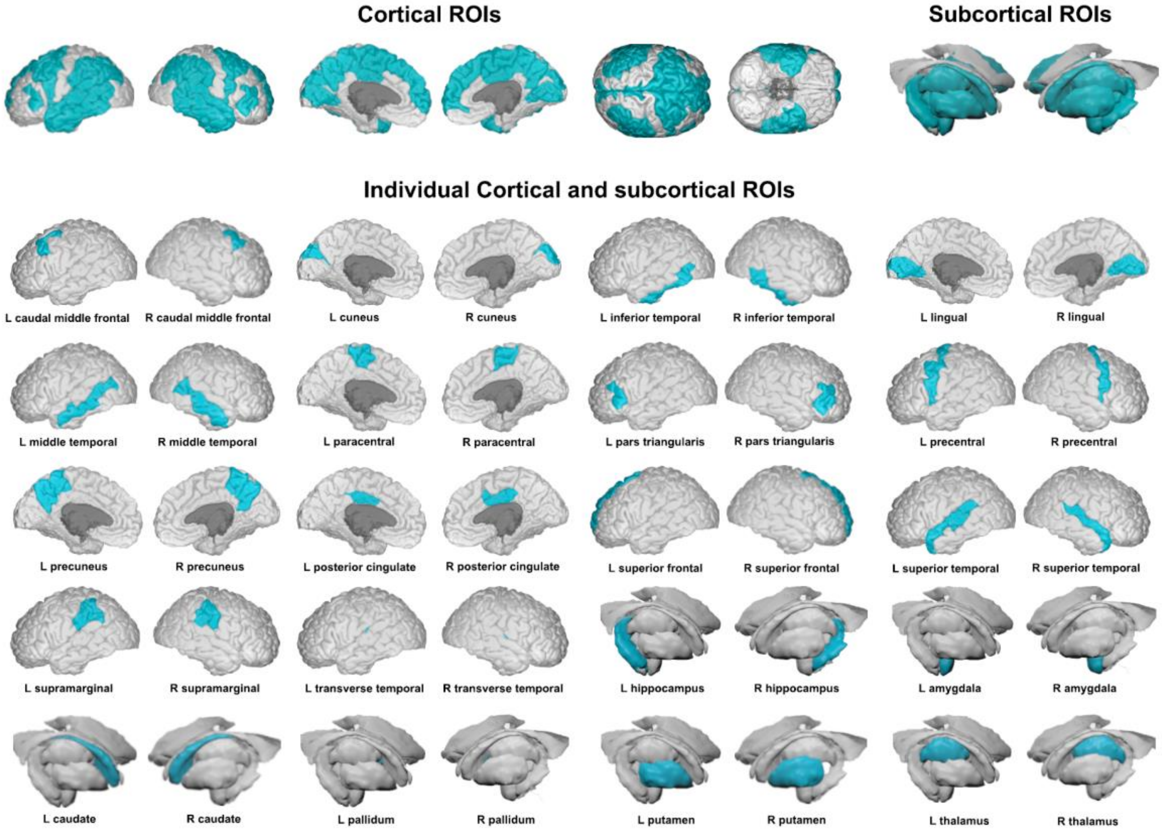

We selected 20 bilateral regions of interest (ROI) of the Desikan-Killiany (DK40) atlas: (a) 14 bilateral cortical regions, including superior frontal gyrus, caudal middle frontal gyrus, inferior frontal gyrus– pars *triangularis*, precentral gyrus, paracentral lobule, superior temporal gyrus, transverse temporal gyrus, middle temporal gyrus, inferior temporal gyrus, supramarginal gyrus, precuneus, posterior cingulate cortex, lingual gyrus, and cuneus; and (b) bilateral ROIs for hippocampus, amygdala, thalamus, and basal ganglia structures, including caudate, globus pallidus, and putamen. The above ROI selection was largely based on the findings of the recent international multicentre ENIGMA-epilepsy structural MRI study.[17] Abbreviations: L= left; R= right; ROI= region of interest.

Supplementary Figure 2.

Individual subtype probability across SuStaIn stage in focal epilepsy of two cohorts.

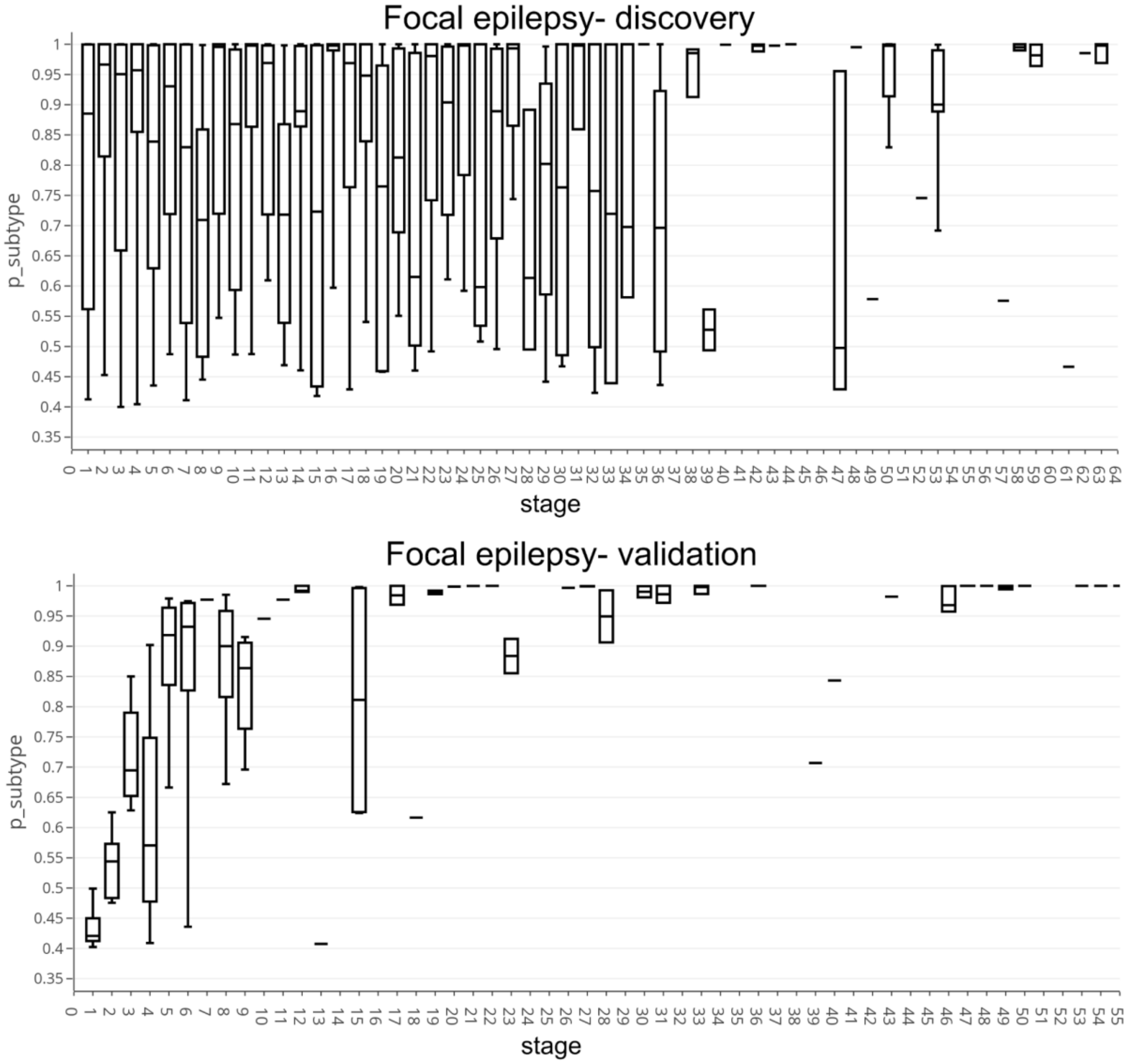

Boxplots representing individual subtype probability across SuStaIn stages are plotted for focal epilepsy-discovery and focal epilepsy validation individuals.

As the duration of illness in focal epilepsy is correlated with SuStaIn stages, Subtype probability was confidently above chance 0.33 (random chance=1/number of subtypes across all non-zero SuStaIn stages, indicating a low likelihood of "cross-over events" or merging events. In focal epilepsy (discovery cohort), the probability was still notably high (>0.90) after stage 47, which closely resembled the findings observed in the validation cohort for focal epilepsy. No evidence was found for putative merging events between the three subtypes after Stage 1.

Supplementary Figure 3.
Progression subtypes in people with unilateral focal epilepsy

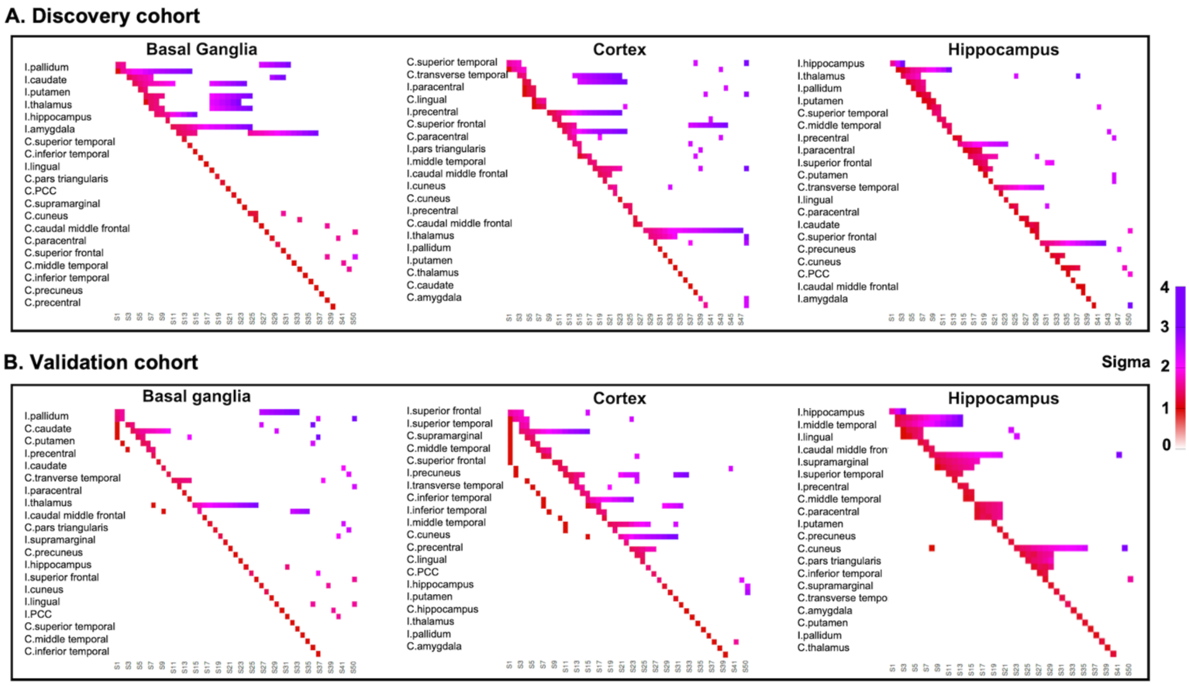

Positional variance diagrams for MRI-based focal epilepsy progression subtypes in the discovery cohort (A) and external validation cohort (B). In both panels, the y-axis shows the most likely sequence of atrophy progression, and the x-axis shows the position of a given region in the progression sequence, with values ranging from one (for the region involved first) to the total number of regions. The intensity of each rectangle corresponds to the proportion of Markov Chain Monte Carlo samples of the posterior distribution whereby a certain region of the y-axis appears at the respective stage of the x-axis. I= ipsilateral; C= contralateral; PCC= posterior cingulate cortex.

Supplementary Figure 4.

Progression subtypes in people with temporal lobe epilepsy and with extra temporal lobe epilepsy

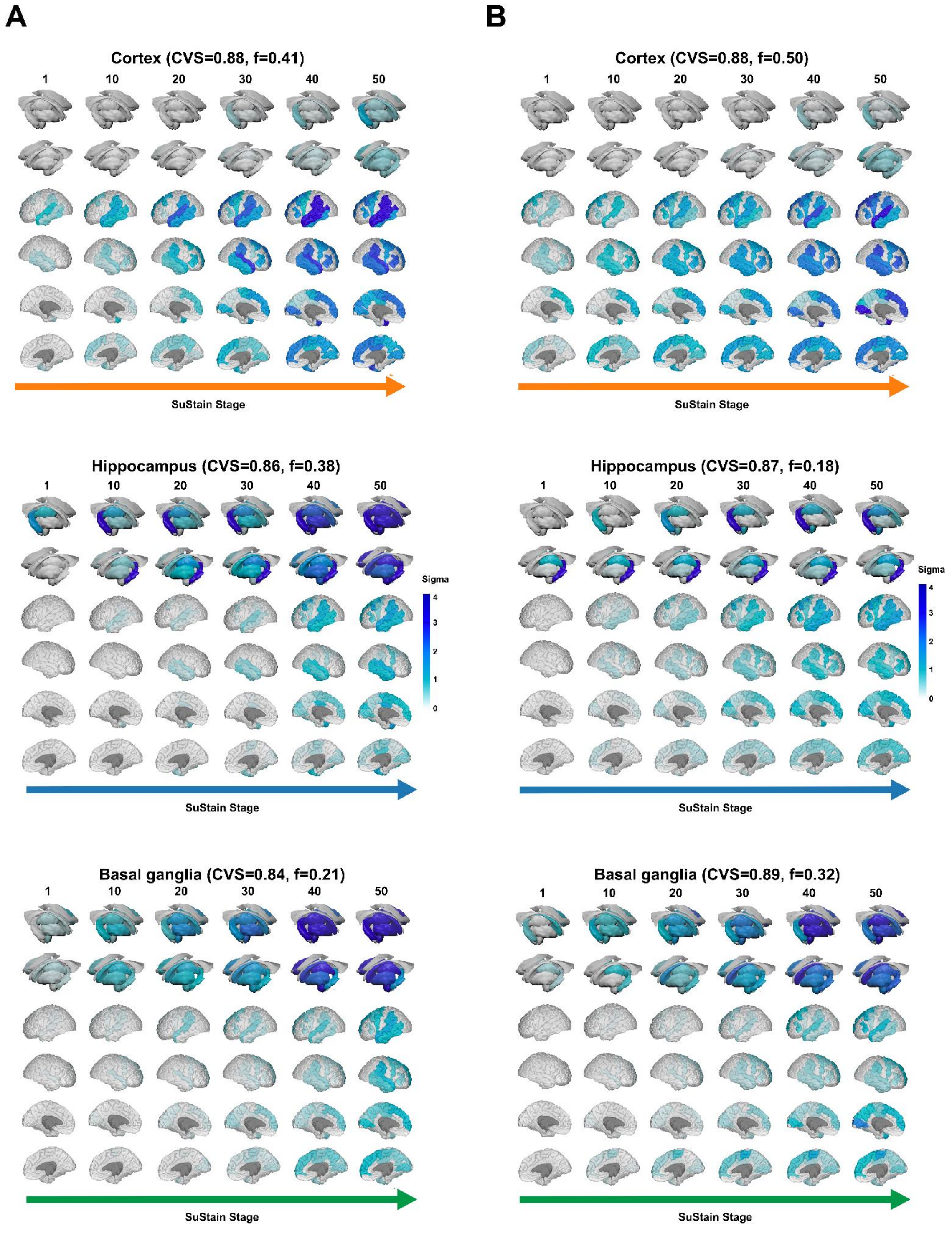

The figure shows the spatiotemporal patterns of progression of grey matter atrophy (subtypes: cortical; basal ganglia; hippocampal) identified via SuStaIn in **A.** temporal lobe epilepsy (TLE) (n=328) and **B.** extratemporal lobe epilepsy (n=175) in the discovery cohort. Each progression pattern in panels consists of a sequence of stages with which cortical thickness and subcortical volumes reach different z-scores in people with epilepsy relative to healthy controls. The colour of each region indicates the severity of grey matter loss; white: unaffected areas; light

blue: mildly affected areas (z-score=1-2); blue: moderately affected areas (z-score=2-3); and dark blue: severely affected areas (z-score >3); “CVS": cross-validation similarity. “f”: proportion of participants assigned to each subtype.

Three similar subtypes based on cortical, basal ganglia (BG), and hippocampal regions were identified. Notably, significant differences between these cohorts in the origin of brain regions in the cortex-led subtype were observed. Specifically, the TLE-only group showed an origin of temporal regions, while the extratemporal lobe epilepsy group displayed an origin of frontal regions. Additionally, the TLE-only group had a greater representation of patients weighted towards the hippocampus-led subtype than the extratemporal lobe epilepsy group (38% vs 18%).

Supplementary Figure 5.

Assignability of the disease subtype with focal epilepsy and IGE in the same cohort

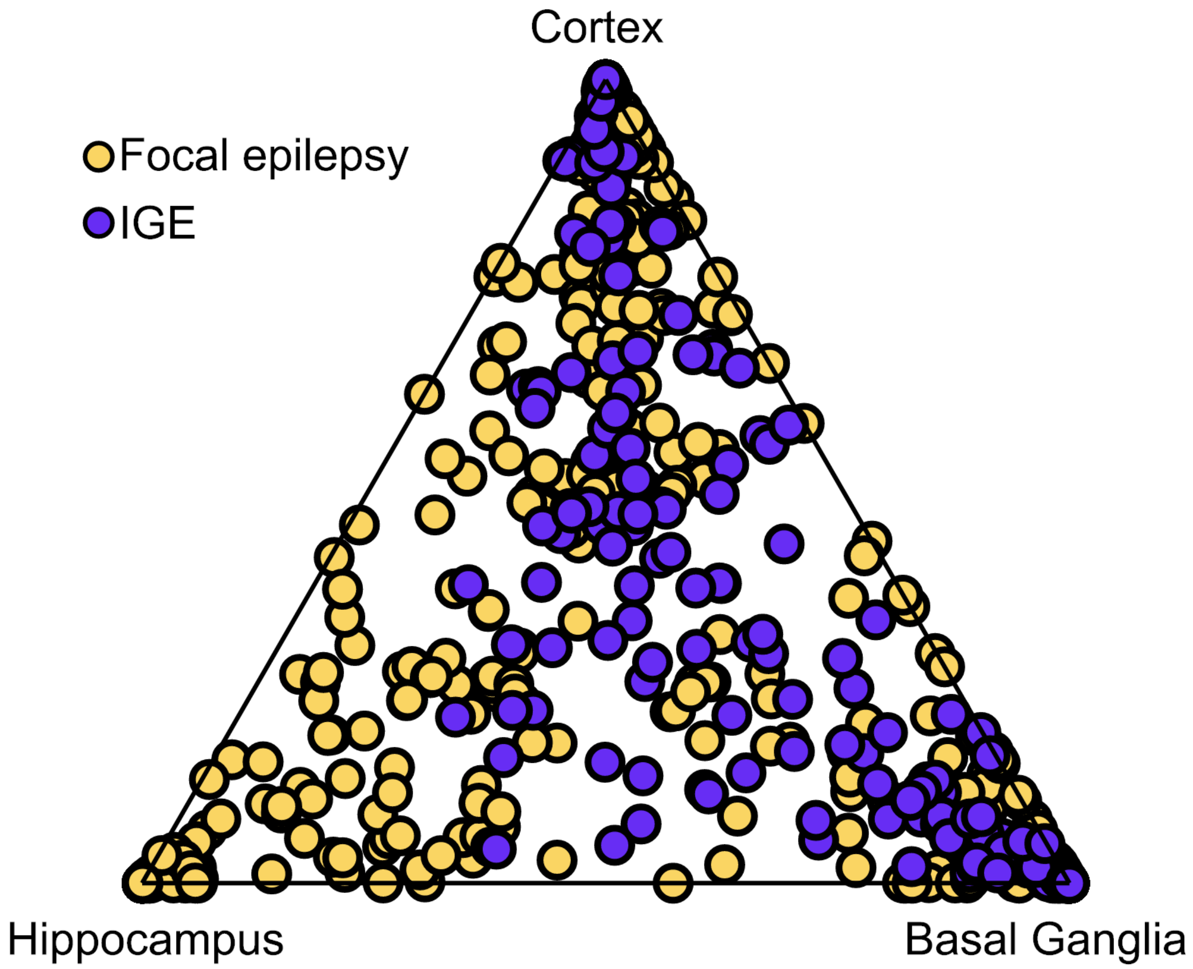

The figure shows the assignability of the disease subtype, operationalised as the distance from each vertex of the triangle, whereby each vertex represents the point at which membership of a given subtype is maximal (100%). Each participant was assigned to one subtype (cortex, basal ganglia, or hippocampus) based on the maximum likelihood of subtype expression. Purple stands for IGE and yellow for focal epilepsy. People with IGE are predominantly represented in the cortex-led and basal ganglia (BG)-led subtypes.
